# A fundamental trade-off among noise suppression, response sensitivity and speed in biological feedback networks

**DOI:** 10.1038/s41540-026-00755-7

**Published:** 2026-06-03

**Authors:** Ka Kit Kong, Feng Liu

**Affiliations:** 1 School of Psychological and Cognitive Sciences, Peking University, Beijing, China; 2 The State Key Laboratory for Artificial Microstructures and Mesoscopic Physics, School of Physics, Peking University, Beijing, China; 3 Key Laboratory of Hebei Province for Molecular Biophysics, Institute of Biophysics, School of Health Science & Biomedical Engineering, Hebei University of Technology, Tianjin, China

**Keywords:** Biophysics, Computational biology and bioinformatics, Mathematics and computing, Physics

## Abstract

Biological regulatory networks rely on feedback control to suppress intrinsic noise while remaining sensitive and responsive to external signals, yet whether these objectives can be achieved simultaneously remains unclear. Here, we show that biological feedback networks face an unavoidable constraint: intrinsic fluctuations cannot be arbitrarily suppressed without sacrificing response sensitivity or slowing response speed via feedback control. Using a general framework for stochastic feedback dynamics, we derive a fundamental trade-off that limits how these three performance objectives can be jointly optimized. Theoretical results and numerical simulations demonstrate that this constraint persists across high-dimensional systems. We further show that nonequilibrium, non-gradient dynamics, which are prevalent in biological regulation, can partially relax but never eliminate this limitation, reducing the minimal cost of noise suppression by at most a factor of two. We validate our theory using a biologically motivated activator–inhibitor feedback motif. Together, our results elucidate a fundamental limitation of feedback control in enhancing the information transmission capacity of biological regulatory networks.

## Introduction

Noise control is critical in signal transmission processes in biological systems. While intrinsic fluctuation due to, among others, the small copy number of molecules and stochastic processes including the birth and death of molecules, is inevitable in a variety of biochemical networks^1–3^, feedback control has been proposed to be a prominent mechanism in suppressing this fluctuation^4–8^.

Moreover, effective signal transmission necessitates not only small fluctuation, but also a high response sensitivity to external signals^9–14^. The relation between intrinsic fluctuation and response sensitivity limits the information transmission capacity of dynamical systems. A potential candidate for this relation could be the fluctuation-dissipation theorem (FDT)^15–21^. Along this line, the energy dissipation of nonequilibrium systems has been proposed to be crucial in attenuating noise while maintaining high sensitivity^22–25^, thus breaking the FDT. For dynamical systems, non-gradient dynamics implies the existence of a rotational flux field, which usually breaks the detailed balance in the steady-state, leading to nonequilibrium^26,27^.

Furthermore, response speed, usually characterized by the dynamic timescale, also plays a crucial role in signal transmission, especially in finite time scenario. For example, it has been proposed that increasing the dynamic timescale can exploit the temporal average effect, breaking the noise-sensitivity proportionality in considering external signal noise^28^. Moreover, the thermodynamic uncertainty relation (TUR) connects the current fluctuation to dissipation in far-from-equilibrium systems, in which slowing down is required to saturate the predicted lower bound for fluctuation^29,30^; and a higher rate of physical processes is usually connected with higher dissipation^9,31^.

Putting these pieces together, although the pure positive or negative feedback has been studied extensively, the feedback implemented in real biological networks are often more complicated^6,32–35^. Yet the general properties of feedback control beyond either positive or negative feedback on these multiple factors remains to be explored^35,36^.

In this study, we evaluate the feedback control mechanism for suppressing intrinsic fluctuation, aiming at disentangling the multiple effects of feedback. We first introduce the theoretical framework, then show the method quantifying the multifaceted effect of feedback and use a one-dimensional system as an illustration. For general high-dimensional feedback control, we further identify a fundamental trade-off among intrinsic fluctuation, response sensitivity, and response speed, indicating that feedback cannot infinitely suppress fluctuation without sacrificing the other two factors. Moreover, we find that the optimal signal transmission set by this trade-off varies with the degree of non-gradient and the effective dimension of the dynamics. Finally, to illustrate the multifaceted effect of feedback, we propose a “control effect diagram” and discuss how this diagram is shaped by the identified trade-off relation.

## Results

### A framework modelling general feedback-controlled networks

**Fig. 1:**
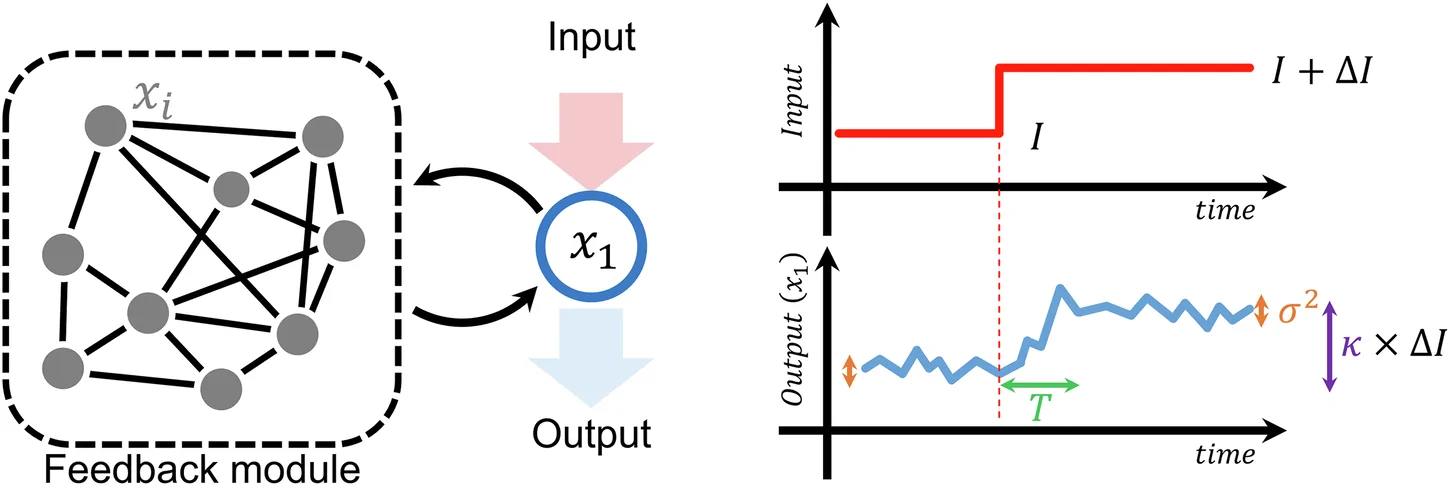
The response fluctuation, sensitivity, and timescale of feedback-controlled networks. The feedback module can be composed of multiple interactive nodes. The number and form of interactions between the feedback module and the response node (${x}_{1}$) is not restricted. The response sensitivity (κ) is defined as the change in the steady-state average of ${x}_{1}$ under a perturbation of the input signal I. The response fluctuation (${\sigma }^{2}$) is defined as the variance of ${x}_{1}$ at the steady-state. The timescale (T) represents the speed of relaxing to the steady-state after a perturbation, which equals the slowest mode of the dynamics.

Biological regulatory networks process external signals in the presence of intrinsic molecular noise, and feedback control is a central mechanism by which such networks modulate their response. To investigate the fundamental constraints imposed by feedback regulation beyond the traditional classified positive or negative feedback, we consider a general class of feedback-controlled signaling networks operating near a unique stable steady-state (Fig. 1). We model the network dynamics starting with a standard Langevin description of stochastic biochemical reactions:

1 $$\frac{d{x}_{i}}{{dt}}={f}_{i}\left(\left\{{x}_{j}\right\},I\right)-{\beta }_{i}{x}_{i}+{\eta }_{i},\,i=1,\ldots ,N,$$

where I is the scalar external input signal (e.g., transcription factor concentration in a gene regulatory network, GRN), ${f}_{i}(\{{x}_{j}\},I)$ is the regulatory function corresponding to the synthesis term of a regulatory network (e.g., transcription in a GRN), ${\beta }_{i}$ is the decay rate (e.g., degradation or dilution), ${\eta }_{i}$ is the white noise term that arises from the intrinsic fluctuation with amplitude: $\langle {\eta }_{i}\left(t\right){\eta }_{j}({t}^{{\prime} })\rangle ={D}_{{ij}}\delta \left(t-{t}^{{\prime} }\right)={D}_{i}{\delta }_{{ij}}\delta \left(t-{t}^{{\prime} }\right)$, and ${D}_{i}=({f}_{i}+{\beta }_{i}{x}_{i})$ following the well-established formulation of the stochasticity in biochemical reactions^37–40^, where $\left\langle \ldots \right\rangle$ denotes the temporal average, $\delta \left(t-{t}^{{\prime} }\right)$ is the Dirac delta function and ${\delta }_{{ij}}$ is the Kronecker delta.

Importantly, our framework places no constraints on the internal organization of the feedback module: the response node ${x}_{1}$ may interact with all other nodes, allowing for complex, multi-step feedback loops that extend beyond simple positive or negative regulation (the feedback module in Fig. 1), mimicking the complex feedback in real biological systems. To balance the complexity of our model, the main signaling pathway is assumed to be consists of a single node, namely the first node in the network, i.e., the external signal only regulate the first node directly (of which the state is ${x}_{1}$), while the other nodes compose a complex feedback module (Fig. 1). Mathematically, this equivalent to constrain $\frac{\partial {f}_{i}}{\partial I}\equiv 0,\,\forall i\ne 1$.

By combining a minimal signaling pathway with a maximally general feedback architecture, this framework enables us to identify constraints that arise from feedback control itself, rather than from specific network topologies or regulatory mechanisms (see Discussion for further justification).

### Definition of the response fluctuation, sensitivity and speed

As an attempt to analytically derive the relation among the three factors, throughout this paper, we consider the dynamical system (Eq. (1)) operates around a unique steady-state, which is defined implicitly by the input I through Eq. (1). Furthermore, for simplicity, following the spirit of linear response theory, we consider the linear response behavior with respect to small perturbation of the input I. We note that extending the modeling for more general nonlinear, multi-stability, or even oscillatory systems remains mathematically challenging and requires further studies.

The response fluctuation is defined as the variance of the response at the steady-state for given input signal, denoted as ${\sigma }^{2}=\langle \delta {x}_{1}^{2}\rangle =\langle {\left({x}_{1}-{x}_{1,{\rm{s}}.{\rm{s}}.}\right)}^{2}\rangle$ (Fig. 1), which can be derived by linearizing the Lyapunov equation^6,39–41^:

2 $${\boldsymbol{J}}{{\boldsymbol{\Sigma }}}_{{\boldsymbol{x}}}+{{\boldsymbol{\Sigma }}}_{{\boldsymbol{x}}}{{\boldsymbol{J}}}^{{\boldsymbol{T}}}+{\boldsymbol{D}}={\bf{0}},$$

where ${{\boldsymbol{\Sigma }}}_{{\boldsymbol{x}}}=\left\langle {\boldsymbol{\delta }}{\boldsymbol{x}}{\boldsymbol{\delta }}{{\boldsymbol{x}}}^{{\boldsymbol{T}}}\right\rangle$ is the covariance matrix at a steady-state, ${\boldsymbol{\delta }}{\boldsymbol{x}}={\left(\delta {x}_{1},\delta {x}_{2},\ldots \right)}^{T}$ is the deviation from the steady-state ${{\boldsymbol{x}}}_{s.s.}={\left({x}_{1,s.s.},\,{x}_{2,s.s.},\,\ldots \right)}^{T}$, ${\boldsymbol{J}}$ is the Jacobian with elements ${J}_{{ij}}={\left(\frac{\partial \left({f}_{i}-{\beta }_{i}{x}_{i}\right)}{\partial {x}_{j}}\right)}_{{x}_{i}={x}_{i,{\rm{s}}.{\rm{s}}.}}$, ${\boldsymbol{D}}$ is the diffusion matrix as defined in Eq. (1). The response noise can be derived from the covariance matrix as: ${\sigma }^{2}\equiv {\sigma }_{1}^{2}\equiv \left\langle \delta {x}_{1}^{2}\right\rangle ={{\boldsymbol{e}}}_{{\bf{1}}}^{{\boldsymbol{T}}}{{\boldsymbol{\Sigma }}}_{{\boldsymbol{x}}}{{\boldsymbol{e}}}_{{\bf{1}}}$, where ${{\boldsymbol{e}}}_{{\bf{1}}}={\left(1,0,\ldots ,0\right)}^{T}$ is the unit vector along the ${x}_{1}$ dimension.

The response sensitivity is defined as the derivative of the steady-state response (${x}_{1,s.s.}$) with respect to the input (I) denoted as $\kappa =\frac{d\left\langle {x}_{1}\right\rangle }{{dI}}$ (Fig. 1), which can be estimated from Eq. (1) near the steady-state:

3 $$\kappa =-{{\boldsymbol{e}}}_{{\bf{1}}}^{{\boldsymbol{T}}}{{\boldsymbol{J}}}^{-{\bf{1}}}{{\boldsymbol{e}}}_{{\bf{1}}}\frac{\partial {f}_{1}}{\partial I}.$$

It is worth mentioning that we focus on the derivative of response, which is equivalent to responding to a small perturbation of the input signal, or the leading order of response with respect to a finite change of the input.

The response speed, throughout this paper, is evaluated using the response timescale, which is defined as the slowest relaxation timescale of the dynamics near the steady-state (Fig. 1), i.e., the inverse of the minimal negative real part of eigenvalues of the Jacobian, denoted as:

4 $$T=\frac{1}{\inf \left(-\mathrm{Re}\left({\lambda }_{i}\right)\right)},$$

where ${\lambda }_{i}$ is the eigenvalues of the Jacobian ${\boldsymbol{J}}$. Since we focus on steady-state response behavior, all eigenvalues of ${\boldsymbol{J}}$ have negative real parts and thus Eq. (4) satisfies T>0.

### Quantification of the effect of feedback control on the fluctuation, sensitivity and speed

In general, the three factors ${\sigma }^{2}$, κ, and T could have multiple degrees of freedom that are *independent* to the feedback control, e.g., the intrinsic fluctuation amplitude ${D}_{1}$, the derivative of synthesis with respect to the input $\frac{\partial {f}_{1}}{\partial I}$, and the decay rate ${\beta }_{1}$ in Eqs. (2), (3), and (4). To isolate and evaluate the contribution of feedback, we comparing these quantities to their ideal values as if no feedback was involved, denoted as ${\sigma }_{0}^{2}$, ${\kappa }_{0}$, and ${T}_{0}$, respectively. For the studied framework (Fig. 1), when the feedback module is absent, the network is reduced to a simple pathway $I\to {x}_{1}$, independent to the original dimension of the system, i.e., the number of nodes in the feedback module. Therefore, the baseline value of ${\sigma }_{0}^{2}$, ${\kappa }_{0}$, and ${T}_{0}$ can be evaluated from a corresponding one-dimensional Langevin equation (with $\frac{\partial {f}_{1}}{\partial {x}_{i}}\equiv 0,\,\forall i$ in Eq. (1)), following:

5 $$\left\{\begin{array}{ccc}{\sigma }_{0}^{2} & = & \frac{{D}_{1}}{2{\beta }_{1}}=\left\langle {x}_{1}\right\rangle \\ {\kappa }_{0} & = & \frac{1}{{\beta }_{1}}\frac{\partial {f}_{1}}{\partial I}\\ {T}_{0} & = & \frac{1}{{\beta }_{1}}\end{array}\right..$$

The effect of feedback on fluctuation, sensitivity and speed is accordingly quantified by the ratios $\frac{{\sigma }^{2}}{{\sigma }_{0}^{2}}$, $\frac{\kappa }{{\kappa }_{0}}$, and $\frac{T}{{T}_{0}}$. Specifically, the effect of feedback on fluctuation equals to the Fano factor $\frac{{\sigma }^{2}}{{\sigma }_{0}^{2}}=\frac{{\sigma }^{2}}{\left\langle {x}_{1}\right\rangle }$. It is worth mentioning that, the definition of the feedback-absent condition in high-dimensional systems necessitate the vanishing of all off-diagonal element in the Jacobian, which can be reduced to the one-dimensional systems discussed.

It should be emphasized that the values of ${D}_{1}$, $\frac{\partial {f}_{1}}{\partial I}$, and, even ${\beta }_{1}$ can generally be different between a feedback system and a feedback-absent system, as there is *no* natural one-to-one correspondence between them. This is why Eq. (5) is necessary to isolate the contribution of feedback: a difference of ${\sigma }^{2}$, κ, and T due to the difference of ${D}_{1}$, $\frac{\partial {f}_{1}}{\partial I}$, and ${\beta }_{1}$ is trivial and is not a unique mechanism of feedback control, while Eq. (5) can quantify these effects and be used to eliminate them (the type-I effect, see Discussion and Fig. 6 for more details). To showcase this rationale, we use a one-dimensional autoregulation system as an example.

### One-dimensional feedback systems

In a one-dimensional system (N=1 in Eq. (1)) with either positive autoregulation or negative autoregulation (depending on the sign of $\frac{\partial {f}_{1}}{\partial {x}_{1}}$), the fluctuation, sensitivity and timescale can be solved from Eqs. (2), (3), and (4) as: ${\sigma }^{2}=\frac{{\beta }_{1}}{{\beta }_{1}-\partial {f}_{1}/\partial {x}_{1}}\left\langle {x}_{1}\right\rangle$, $\kappa =\frac{1}{{\beta }_{1}-\partial {f}_{1}/\partial {x}_{1}}\frac{\partial {f}_{1}}{\partial I}$, and $T=\frac{1}{{\beta }_{1}-\partial {f}_{1}/\partial {x}_{1}}$. Comparing these results to Eq. (5), if, say, timescale is reduced ($T < {T}_{0}$) because of a significant difference of ${\beta }_{1}$ between the two systems, no conclusion can be drawn about how the autoregulation (${x}_{1}\to {x}_{1}$) affects the response speed. Therefore, all parameters in Eq. (5) are assumed to be identical to that in the feedback system, which than yields:

6 $$\frac{{\sigma }^{2}}{{\sigma }_{0}^{2}}=\frac{\kappa }{{\kappa }_{0}}=\frac{T}{{T}_{0}}=\frac{{\beta }_{1}}{{\beta }_{1}-\partial {f}_{1}/\partial {x}_{1}},$$

leading to the well-known properties that negative feedback ($\frac{\partial {f}_{1}}{\partial {x}_{1}} < 0$) can suppress fluctuation ($\frac{{\sigma }^{2}}{{\sigma }_{0}^{2}} < 1$) and speed up the response ($\frac{T}{{T}_{0}} < 1$)^4,5^. Based on this framework, Eq. (6) also implies that fluctuation suppression ($\frac{{\sigma }^{2}}{{\sigma }_{0}^{2}} < 1$) always requires the same fold of sensitivity reduction in 1D systems ($\frac{\kappa }{{\kappa }_{0}}=\frac{{\sigma }^{2}}{{\sigma }_{0}^{2}} < 1$), resembling one classic prediction of the FDT: the steady-state responding to a conjugate signal perturbation is proportional to its intrinsic fluctuation, i.e., $\kappa \propto {\sigma }^{2}$. It is worth emphasizing again that this conclusion is based on Eq. (6), which is valid only if all variables ($\left\langle {x}_{1}\right\rangle ,\,{\beta }_{1},\frac{\partial {f}_{1}}{\partial {x}_{1}},\frac{\partial {f}_{1}}{\partial I}$) are identical between the feedback-controlled and feedback-absent systems.

There is, however, no guarantee that the relation of Eq. (6) can be applied to high-dimensional systems, it remains unclear how general feedback affects the response sensitivity, and whether a relation between fluctuation, sensitivity, and timescale exists.

For high dimensional systems, to further guarantee the comparability between the feedback and feedback-absent system, in addition to equalize all parameters in Eq. (5) to the feedback systems, no additional source of fluctuation is considered in the feedback module, i.e., ${D}_{i}=0,\,\forall i\ne 1$ in Eq. (1), since this fluctuation does not exist in the feedback-absent condition (the type-II effect, see Discussion and Fig. 6 for more details). Relaxing this constraint does not affect our main conclusion as a lower bound for fluctuation remains when additional independent fluctuation is introduced (see Discussion for more details).

It is worth mentioning that a similar protocol has been proposed recently in studying the role of feedback in adaptation^35,36^.

### A trade-off among fluctuation, sensitivity, and speed in feedback-controlled networks

Based on the introduced scheme, we first focus on the potential relation between fluctuation and sensitivity for 2D systems, aiming at an FDT-like relation. Under this setup, solving Eqs. (2) and (3) yields (Supplementary Note 1):

7 $$\left\{\begin{array}{ccc}{\sigma }^{2} & = & -{D}_{1}\frac{{J}_{22}^{2}+\Delta }{2\Delta \tau }\\ \kappa & = & -\frac{\partial {f}_{1}}{\partial I}\frac{{J}_{22}}{\Delta }\end{array}\right.,$$

where $\Delta =\det \left({\boldsymbol{J}}\right) > 0$ is the determinant of the Jacobian, and $\tau ={\rm{tr}}\left({\boldsymbol{J}}\right) < 0$ is the trace of the Jacobian. Comparing to the feedback-absent condition (Eq. (5)), we find $\frac{{\sigma }^{2}}{{\sigma }_{0}^{2}}/\left|\frac{\kappa }{{\kappa }_{0}}\right|\ge 2\frac{\sqrt{T/{T}_{\min }}}{1+T/{T}_{\min }}$ (Supplementary Note 1), where ${T}_{\min }$ is the fastest timescale of the dynamics defined based on the Jacobian. This inequality, however, implies that *no* fundamental lower bound exists for $\frac{{\sigma }^{2}}{{\sigma }_{0}^{2}}$ given sensitivity $\left|\frac{\kappa }{{\kappa }_{0}}\right|$, as long as the system exhibits a timescale separation $\frac{T}{{T}_{\min }}\gg 1$ (Supplementary Fig S1(a)), indicating fluctuation can be, theoretically, suppressed infinitely without affecting the sensitivity through a feedback control in 2D systems. This conclusion is extensible to higher dimensional systems, which is also numerically validated (Supplementary Fig S1(b)).

Though there is no fundamental constraint between fluctuation and sensitivity for 2D systems, combining the effect of feedback on the timescale (Eqs. (4) and (5)), we derive a closed-form three-way trade-off among fluctuation, sensitivity, and timescale following (Supplementary Note 1):

8 $$\frac{{\sigma }^{2}}{{\sigma }_{0}^{2}}\frac{T}{{T}_{0}}/{\left(\frac{\kappa }{{\kappa }_{0}}\right)}^{2} > \frac{1}{2}.$$

**Fig. 2:**
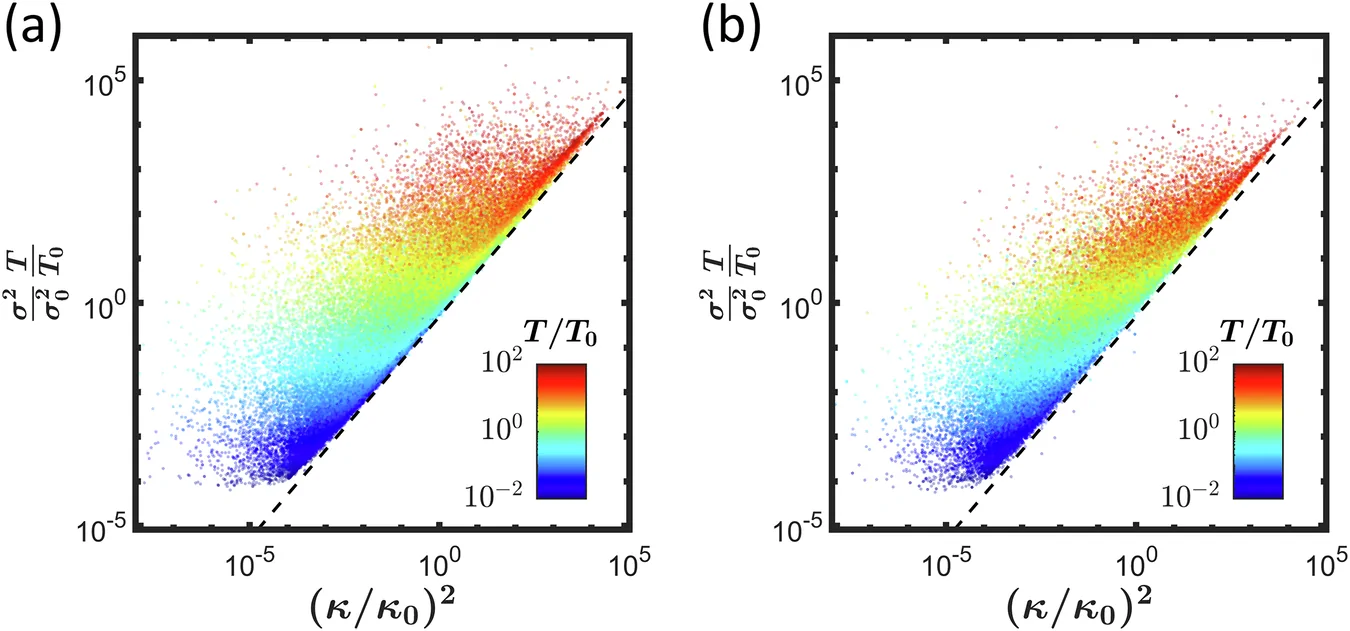
The fluctuation-sensitivity-timescale trade-off in 2D and 10D dynamical systems. Numerical results of parameter sampling in 2D (**a**) and 10D (**b**) dynamics, in which the feedback module is composed of 1 and 9 nodes, respectively (Supplementary Note 4). The results show that the predicted lower bound (Eq. (8)) is reachable (dashed line). Each dot is the result of a sampled parameter set.

The numerical results of 2D systems based on the nonlinear Monod-Wyman-Changeux (MWC) model^42–45^ with 100,000 randomly sampled parameter sets suggest that this bound can be reached in certain parameters (Fig. 2(a) and Supplementary Note 4). For systems with higher dimensions, numerical results show that the trade-off relation (Eq.(8)) is still valid and reachable (Fig. 2(b)), suggesting that Eq. (8) is a fundamental trade-off for dynamics following the feedback-controlled framework in Fig. 1.

While the variables (${\sigma }^{2}$, κ, T) quantify the dynamics properties of the system, the ratios ($\frac{{\sigma }^{2}}{{\sigma }_{0}^{2}}$, $\frac{\kappa }{{\kappa }_{0}}$, $\frac{T}{{T}_{0}}$) quantify the *effect* of feedback. Accordingly, this trade-off reveals that, by feedback control, the fluctuation can only be suppressed up to half without affecting either the sensitivity or the timescale, i.e., $\frac{{\sigma }^{2}}{{\sigma }_{0}^{2}} > \frac{1}{2}$ when $\frac{\kappa }{{\kappa }_{0}}=1$ and $\frac{T}{{T}_{0}}=1$; further suppression of fluctuation ($\frac{{\sigma }^{2}}{{\sigma }_{0}^{2}} < \frac{1}{2}$) always sacrifices the response sensitivity ($\frac{\kappa }{{\kappa }_{0}} < 1$) or response speed ($\frac{T}{{T}_{0}} > 1$); infinite suppression of fluctuation ($\frac{{\sigma }^{2}}{{\sigma }_{0}^{2}}\to 0$) without affecting the sensitivity ($\frac{\kappa }{{\kappa }_{0}}=1$) generally requires infinitely slow dynamics ($\frac{T}{{T}_{0}}\to \infty$). This result indicates a trilemma for the effect of general feedback control. Although the lower bound appears to follow ${\sigma }^{2}\propto {\kappa }^{2}$, it cannot be explained by the error propagation, since the source of fluctuation considered here is intrinsic to the system rather than inherited from the input signal.

### The optimal performance is bound by the degree of non-gradient

While the trade-off (Eq. (8)) is valid for high dimensional systems defining a lower bound for $\frac{{\sigma }^{2}}{{\sigma }_{0}^{2}}\frac{T}{{T}_{0}}/{\left(\frac{\kappa }{{\kappa }_{0}}\right)}^{2}$ being $\frac{1}{2}$, 1D systems exhibit an apparently worse performance following $\frac{{\sigma }^{2}}{{\sigma }_{0}^{2}}\frac{T}{{T}_{0}}/{\left(\frac{\kappa }{{\kappa }_{0}}\right)}^{2}=1$ (from Eq. (6)). This may result from the nature that 1D dynamics are always gradient. To test this hypothesis, we next focus on high-dimensional gradient systems.

Since the eigen dimensions of a gradient system evolve independently under small perturbation, solving ${\sigma }^{2}$, κ, and T becomes tractable for arbitrary dimensions. We find that for high dimensional gradient systems, the trade-off relation is altered (Supplementary Note 2):

9 $$\frac{{\sigma }^{2}}{{\sigma }_{0}^{2}}\frac{T}{{T}_{0}}/{\left(\frac{\kappa }{{\kappa }_{0}}\right)}^{2}\ge 1.$$

The lower bound is uplifted to 1, indicating that the suppression of fluctuation ($\frac{{\sigma }^{2}}{{\sigma }_{0}^{2}} < 1$) *always* sacrifices response sensitivity ($\frac{\kappa }{{\kappa }_{0}} < 1$) or response speed ($\frac{T}{{T}_{0}} > 1$). This result implies that gradient systems perform worse than non-gradient systems regarding signal transmission. Numerical results of local gradient systems support that this closed-form is a tight bound in both 2D and higher-dimensional (10D as an example) dynamics (Supplementary Fig S2).

So far, we have found that the lower bound for $\frac{{\sigma }^{2}}{{\sigma }_{0}^{2}}\frac{T}{{T}_{0}}/{\left(\frac{\kappa }{{\kappa }_{0}}\right)}^{2}$ in gradient and non-gradient systems is 1 and $\frac{1}{2}$, respectively. Next, we investigate how this bound relies on the degree of non-gradient of a dynamical system. However, as far as we know, no common method exists to directly quantify the degree of non-gradient using a single scalar, hence we adopt one based on the symmetric-antisymmetric decomposition of the Jacobian.

The Jacobian of a dynamical system (Eq. (1)) near a steady-state can always be decomposed into a symmetric and an antisymmetric part, i.e., ${\boldsymbol{J}}={{\boldsymbol{J}}}^{{\bf{s}}}{\boldsymbol{+}}{{\boldsymbol{J}}}^{{\bf{as}}}$, where the symmetric part ${{\boldsymbol{J}}}^{{\bf{s}}}=\frac{\left({\boldsymbol{J}}+{{\boldsymbol{J}}}^{{\boldsymbol{T}}}\right)}{2}$ represents a gradient component, and the antisymmetric part ${{\boldsymbol{J}}}^{{\bf{as}}}=\frac{\left({\boldsymbol{J}}-{{\boldsymbol{J}}}^{{\boldsymbol{T}}}\right)}{2}$ represents a rotational component. For a gradient field determined by ${{\boldsymbol{J}}}^{{\bf{s}}}$, the eigen dimension with the maximal eigenvalue ${\lambda }_{1}\left({{\boldsymbol{J}}}^{{\bf{s}}}\right)$ defines its stability and is decisive: if ${\lambda }_{1}\left({{\boldsymbol{J}}}^{{\bf{s}}}\right) < 0$, ${{\boldsymbol{J}}}^{{\bf{s}}}$ represents a stable dynamic, whose relaxation timescale is given by ${\left(-{\lambda }_{1}\left({{\boldsymbol{J}}}^{{\bf{s}}}\right)\right)}^{-1}$; if ${\lambda }_{1}\left({{\boldsymbol{J}}}^{{\bf{s}}}\right)\ge 0$, ${{\boldsymbol{J}}}^{{\bf{s}}}$ represents an unstable dynamic, even though ${\boldsymbol{J}}$ can represent stable dynamics, and ${\lambda }_{1}\left({{\boldsymbol{J}}}^{{\bf{s}}}\right)$ corresponds to the most unstable dimension and defines its reactivity^46^. For a rotational field determined by ${{\boldsymbol{J}}}^{{\bf{as}}}$, whose eigenvalues are all pure imaginary, the eigenvalue with the maximal imaginary part ${\lambda }_{1}\left({{\boldsymbol{J}}}^{{\bf{as}}}\right)$ determines the maximal flux speed as ${\rm{im}}\left({\lambda }_{1}\left({{\boldsymbol{J}}}^{{\bf{as}}}\right)\,\right) > 0$. Accordingly, we quantify the degree of non-gradient by a non-negative scalar χ defined as:

10 $$\chi =\frac{{\rm{im}}\left({\lambda }_{1}\left({{\boldsymbol{J}}}^{{\bf{as}}}\right)\right)}{{\rm{im}}\left({\lambda }_{1}\left({{\boldsymbol{J}}}^{{\bf{as}}}\right)\right)-{\lambda }_{1}\left({{\boldsymbol{J}}}^{{\bf{s}}}\right)}.$$

A large value of χ indicates a potentially high degree of non-gradient. For gradient systems, ${\rm{im}}\left({\lambda }_{1}\left({{\boldsymbol{J}}}^{{\bf{as}}}\right)\right)=0$ and thus χ=0; for systems with $-{\lambda }_{1}\left({{\boldsymbol{J}}}^{{\bf{s}}}\right)\,\gg \,\mathrm{im}\left({\lambda }_{1}\left({{\boldsymbol{J}}}^{{\bf{as}}}\right)\right) > 0$, its gradient part is stable and dominant, thus the degree of non-gradient is small, i.e., $\chi \sim 0$; for systems with ${\lambda }_{1}\left({{\boldsymbol{J}}}^{{\bf{s}}}\right)\gg 0$, its gradient part is highly unstable and the non-gradient part is necessary to stabilize the system, implying a high degree of non-gradient and χ>1.

Based on this quantification and combining the expression of ${\sigma }^{2}$, κ, T, and their corresponding baseline value (Eqs. (4), (5), and (7)), we find that for 2D systems, the lower bound for $\frac{{\sigma }^{2}}{{\sigma }_{0}^{2}}\frac{T}{{T}_{0}}/{\left(\frac{\kappa }{{\kappa }_{0}}\right)}^{2}$ depends on χ, following (Supplementary Note 3):

11 $$\frac{{\sigma }^{2}}{{\sigma }_{0}^{2}}\frac{T}{{T}_{0}}/{\left(\frac{\kappa }{{\kappa }_{0}}\right)}^{2}\ge {{\mathcal{B}}}_{{\rm{G}}}\left(\chi \right)=\frac{1}{2}\left[1+{\left(\frac{1}{1+\chi }\right)}^{2}\right].$$

**Fig. 3:**
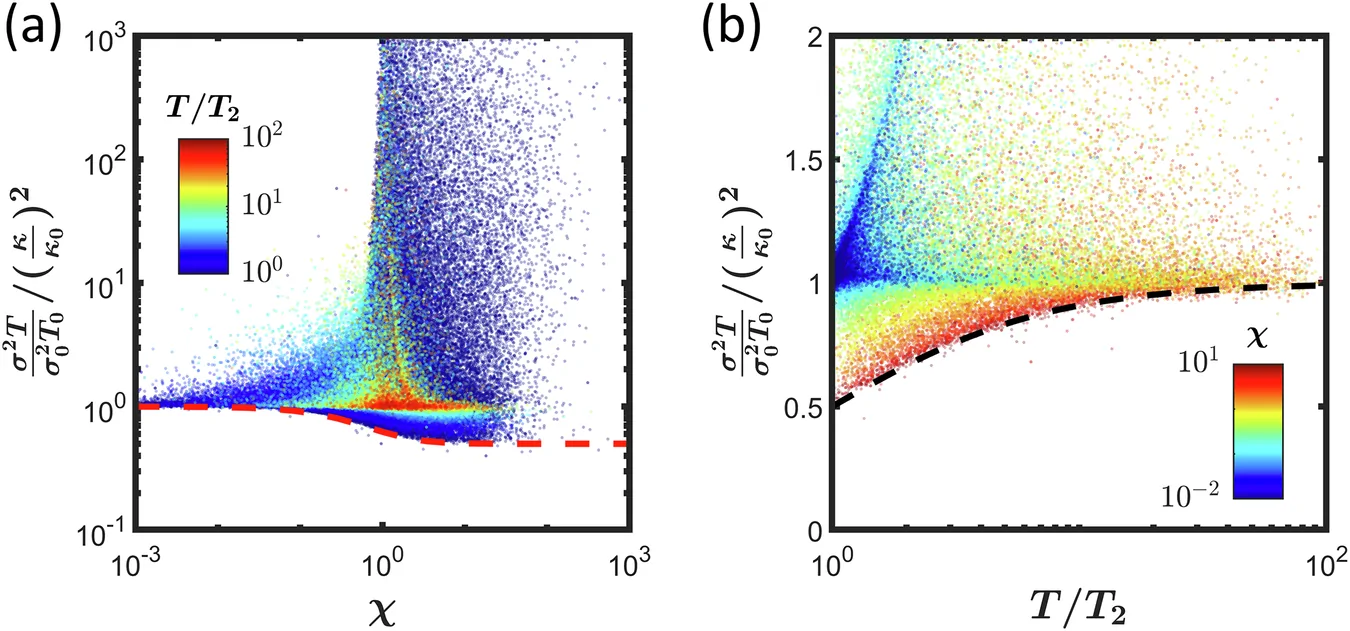
The effect of non-gradient and effective dimention on the trade-off relation. The lower bound for $\frac{{\sigma }^{2}}{{\sigma }_{0}^{2}}\frac{T}{{T}_{0}}/{\left(\frac{\kappa }{{\kappa }_{0}}\right)}^{2}$ is determined by the degree of non-gradient χ (Eq. (10)) (**a**) and the ratio between the two slowest timescales $T/{T}_{2}$ (**b**) in 2D systems. Numerical results of 2D systems show that the predicted lower bound in Eqs. (11) and (12) (dashed curve in (**a**) and (**b**), respectively) are tight lower bounds.

For gradient systems, the lower bound is ${{\mathcal{B}}}_{{\rm{G}}}\left(0\right)=1$; for systems with a high degree of non-gradient, ${{\mathcal{B}}}_{{\rm{G}}}\left(+\infty \right)\to \frac{1}{2}$. Moreover, ${{\mathcal{B}}}_{{\rm{G}}}\left(\chi \right)$ is a monotonically decreasing function, implying that a high level of non-gradient always improves the optimal performance. Numerical results with exhaustive parameter sampling support the validity of Eq. (11) for both 2D systems (Fig. 3(a)) and high-dimensional systems (Supplementary Fig S3(a)).

Intuitively, the eigen dimensions of a gradient system evolve independently, mimicking multiple one-dimensional dynamics with a worse lower bound. The dynamics of the whole system becomes a direct sum of these components without interaction among them, thus leading to a worse performance comparing to general non-gradient systems.

It is worth noting that the decomposition of dynamical systems into a gradient and a non-gradient field is not unique, others including decomposition based on the steady-state distribution, the Helmholtz decomposition, etc.^26,47,48^. We use the symmetric-antisymmetric decomposition as it depends explicitly on the Jacobian and thus facilitates analytical derivation. Moreover, the rotational component of the symmetric-antisymmetric decomposition is always a curl field when projected onto any 2D subspace in which the curl operator is well-defined.

### The lower bound for the trade-off is affected by the effective dimension

While the dimension of the *de facto* trajectory of a dynamical system is usually lower than that of the whole variable space, it is critical to characterize the effective dimension of the actual dynamics because a lower bound for the trade-off less than 1 necessitates a dimension higher than 1. To have an effective dimension higher than 1 requires, at least, the two slowest modes to occur at a similar timescale. This condition can be quantified by a ratio $T/{T}_{2}$, where T is the slowest timescale as defined before, and ${T}_{2}$ is the second slowest timescale. In 2D systems, we find a lower bound for $\frac{{\sigma }^{2}}{{\sigma }_{0}^{2}}\frac{T}{{T}_{0}}/{\left(\frac{\kappa }{{\kappa }_{0}}\right)}^{2}$ depending on $T/{T}_{2}$ (Supplementary Note 3):

12 $$\frac{{\sigma }^{2}}{{\sigma }_{0}^{2}}\frac{T}{{T}_{0}}/{\left(\frac{\kappa }{{\kappa }_{0}}\right)}^{2} > {{\mathcal{B}}}_{{\rm{D}}}\left(\frac{T}{{T}_{2}}\right)=\frac{T/{T}_{2}}{1+T/{T}_{2}},$$

where ${B}_{{\rm{D}}}\left(\frac{T}{{T}_{2}}\right)\ge \frac{1}{2}$ since $T\ge {T}_{2}$. Equation (12) implies that when $\frac{T}{{T}_{2}}\to +\infty$, the lower bound ${B}_{{\rm{D}}}\left(+\infty \right)\to 1$, which is reasonable since this timescale separation implies that the central manifold of the dynamic is effectively 1D. When $\frac{T}{{T}_{2}}\sim 1$, the two underlying dimensions are on the same timescale, allowing the system to exploit a 2D phase space and achieve a lower bound ${B}_{{\rm{D}}}\left(1\right)=\frac{1}{2}$. Numerical results with exhaustive parameter sampling support the validity of Eq. (12) for both 2D systems (Fig. 3(b)) and high dimensional systems (Supplementary Fig. S3(b)).

Equations (11) and (12) appear to be two different bounds for the trade-off relating to different dynamical properties of the system, they are, however, not completely independent (Fig. 3). While a large χ ensures the potential for a high degree of non-gradient, a small $\frac{T}{{T}_{2}}$ permits the dynamical trajectory to exploit higher dimensional space and, thus, the degree of non-gradient.

### Illustrating the multifaceted effects of feedback control

So far, we have generally discussed the effect of feedback control on fluctuation, sensitivity and timescale by exploiting the technique of linear response. To make our result more tangible, we test the theoretical prediction on a more realistic biological model. We consider an adopted activator-inhibitor motif with strong nonlinearity as an example, where the activator is receiving an external input and act as the response node (blue node in Fig. 5(c)). In this system, the auto-activation of the activator and the feedback from the inhibitor together form a complex feedback control. Since an auto-inhibition of the inhibitor is introduced in the model, the feedback from the inhibitor is neither positive nor negative, making this system a complex feedback system beyond the classic dichotomous classification. Moreover, the Hill function, the usual regulatory function used in the biological networks, is implemented in this example. In this system, the “concentration” of the activator (${x}_{1}$) and inhibitor (${x}_{2}$) evolve following:

13 $$\left\{\begin{array}{ccc}\frac{d{x}_{1}}{{dt}} & = & {\alpha }_{I}\frac{{I}^{{n}_{I}}}{{I}^{{n}_{I}}+{K}_{I}^{{n}_{I}}}+{\alpha }_{11}\frac{{x}_{1}^{{n}_{11}}}{{x}_{1}^{{n}_{11}}+{K}_{11}^{{n}_{11}}}+{\alpha }_{12}\frac{{K}_{12}^{{n}_{12}}}{{x}_{2}^{{n}_{12}}+{K}_{12}^{{n}_{12}}}-{\beta }_{1}{x}_{1}+{\eta }_{1}\\ \frac{d{x}_{2}}{{dt}} & = & \,{\alpha }_{21}\frac{{x}_{1}^{{n}_{21}}}{{x}_{1}^{{n}_{21}}+{K}_{21}^{{n}_{21}}}+{\alpha }_{22}\frac{{K}_{22}^{{n}_{22}}}{{x}_{2}^{{n}_{22}}+{K}_{22}^{{n}_{22}}}-{\beta }_{2}{x}_{2}\end{array}\right.,$$

where $\left\langle {\eta }_{1}\left(t\right){\eta }_{1}\left(t^{\prime} \right)\right\rangle =\frac{1}{{C}_{1}}\left({\alpha }_{I}\frac{{I}^{{n}_{I}}}{{I}^{{n}_{I}}+{K}_{I}^{{n}_{I}}}+{\alpha }_{11}\frac{{x}_{1}^{{n}_{11}}}{{x}_{1}^{{n}_{11}}+{K}_{11}^{{n}_{11}}}+{\alpha }_{12}\frac{{K}_{12}^{{n}_{12}}}{{x}_{2}^{{n}_{12}}+{K}_{12}^{{n}_{12}}}+{\beta }_{1}{x}_{1}\right)\delta \left(t-{t}^{{\prime} }\right)$ following the usual chemical Langevin equation, and ${C}_{1}=100$ is the same as the previous parameter sampling in Fig. (2) (Supplementary Note 4), making the noise amplitude of this simulation comparable to previous results. Following the introduced scheme, ${x}_{2}$ is assumed to be noise-free, corresponding to a perfect feedback control.

**Fig. 4:**
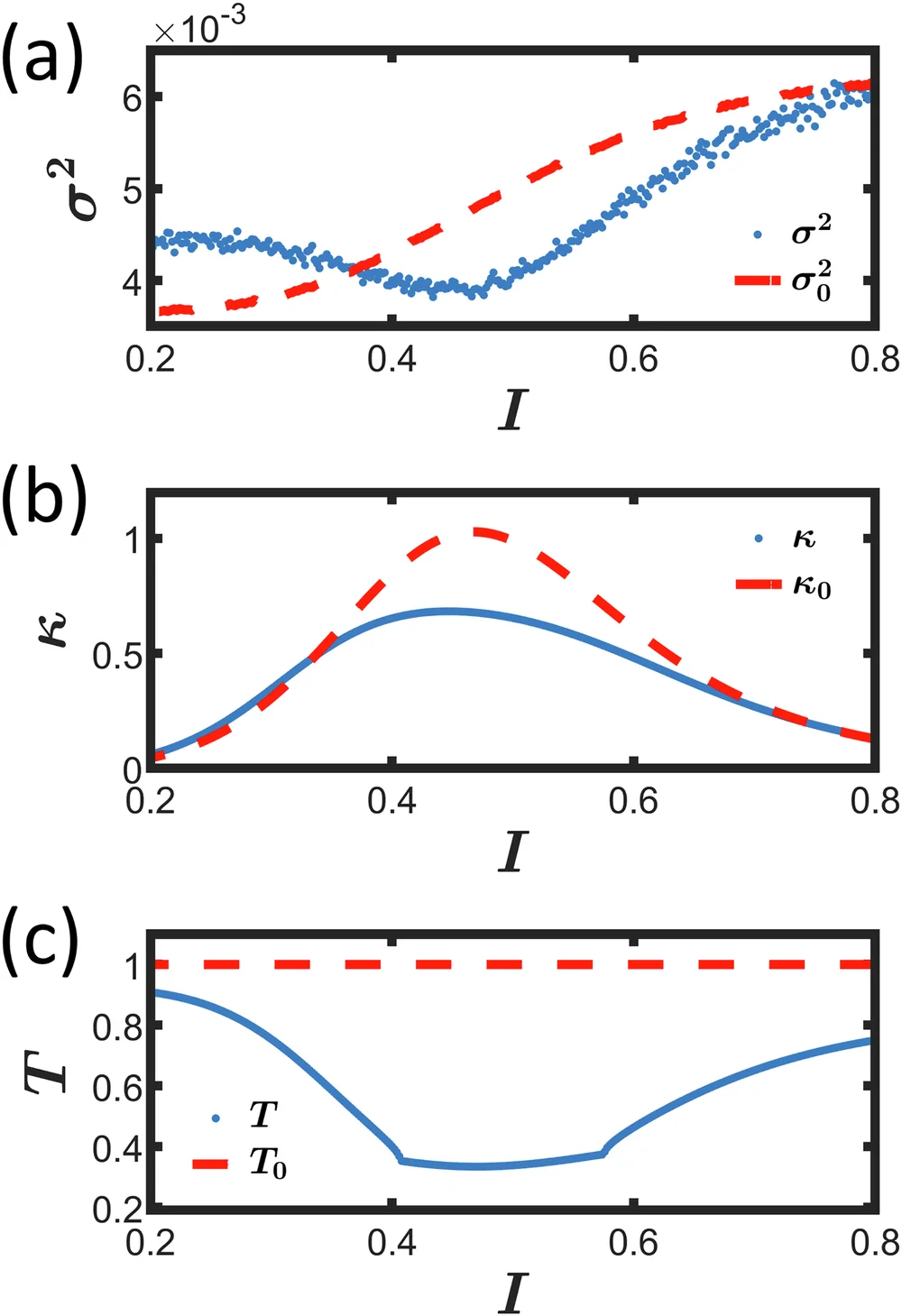
The interwinding effect of feedback on resposne fluctuation, sensitivity, and timescale. In a biological feedback network with an adopted activator-inhibitor motif, the effect of feedback on response fluctuation (**a**), sensitivity (**b**), and timescale (**c**) is generally coupled and nonmonotonic. For the given parameters, in low level of input (I), the noise is amplified by the feedback while sensitivity is slightly enhanced, and it is the opposite in high level of I; while the response speed is enhanced though the whole simulated range of I.

**Fig. 5:**
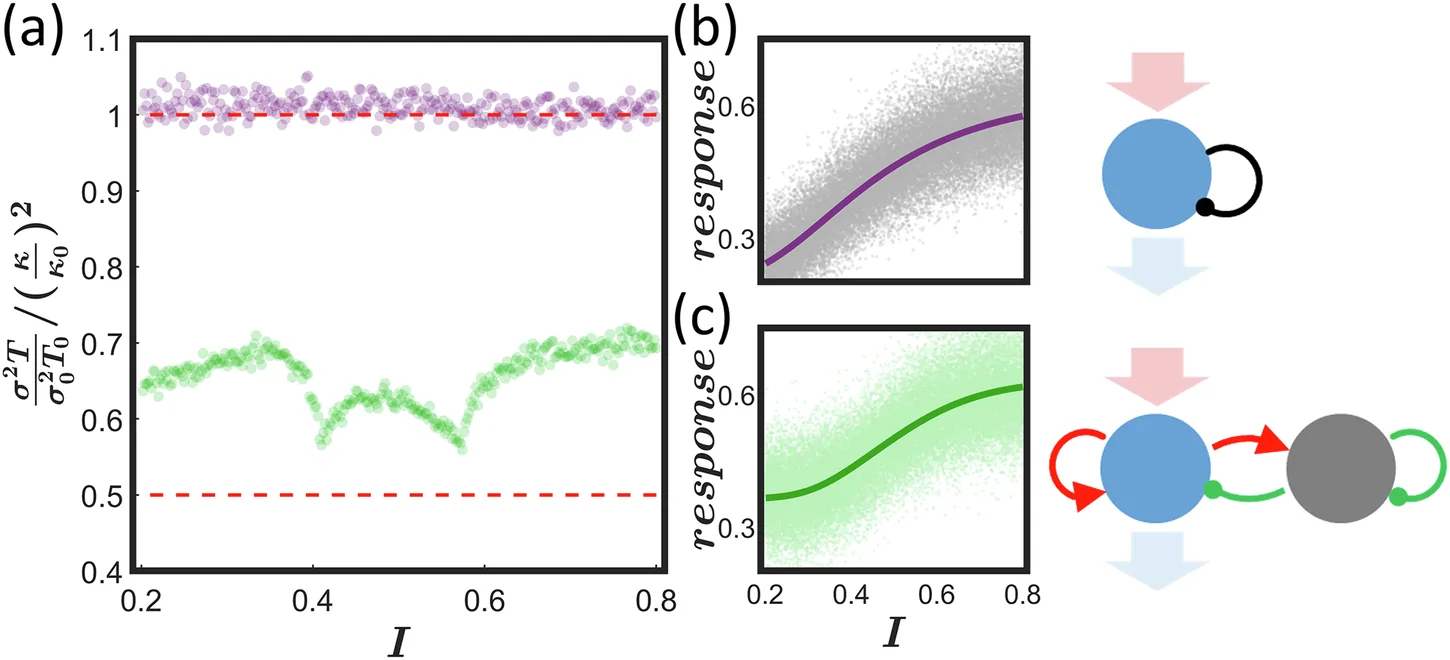
The overall effect of feedback control in the biological feedback network captured by the product $\frac{{\sigma }^{2}}{{\sigma }_{0}^{2}}\frac{T}{{T}_{0}}/{\left(\frac{\kappa }{{\kappa }_{0}}\right)}^{2}$ . **a** The product is between $\frac{1}{2}$ and 1 (the two red dashed line) in the complex feedback systems (the adopted activator-inhibitor motif, green dots) but equals 1 in simple auto-inhibition system (purple dots). The response curves and network motifs of the auto-inhibition (**b**) and complex feedback (**c**) systems.

Simulation results (Fig. 4) show that, comparing to the feedback-absent condition (Eq. (5)), feedback control tunes the three factors (${\sigma }^{2}$, κ, and T) simultaneously with varying, nonmonotonic amplitude across different input level (I). For the given parameters, the response speed is enhanced over the whole range of input ($\frac{T}{{T}_{0}} < 1$, Fig. 4(c)), and stronger at the middle level. At low I, the response sensitivity is also slightly enhanced ($\frac{\kappa }{{\kappa }_{0}} > 1$, Fig. 4(b)) at the cost of amplifying fluctuation ($\frac{{\sigma }^{2}}{{\sigma }^{2}} > 1$, Fig. 4(b)); while this effect is turnover at higher I, the response sensitivity is sacrificed ($\frac{\kappa }{{\kappa }_{0}} < 1$) for the suppression the fluctuation ($\frac{{\sigma }^{2}\,}{{\sigma }_{0}^{2}} < 1$). These rich effects of feedback cannot be comprehensively understood from the perspective of simple positive or negative feedback. With these individual effects being quantified, the overall contribution of the feedback can be captured by the product $\frac{{\sigma }^{2}}{{\sigma }_{0}^{2}}\frac{T}{{T}_{0}}/{\left(\frac{\kappa }{{\kappa }_{0}}\right)}^{2}$. The results (Fig. 5) show that the value of this product can achieve a value of less than 1 for the whole range of input, but no less than $\frac{1}{2}$. As a comparison, the product of a simple auto-inhibition systems always equals to 1 (Fig. 5, Eq. (6)). These results together illustrate the interwinding effects of complex feedback on the three factors.

**Fig. 6:**
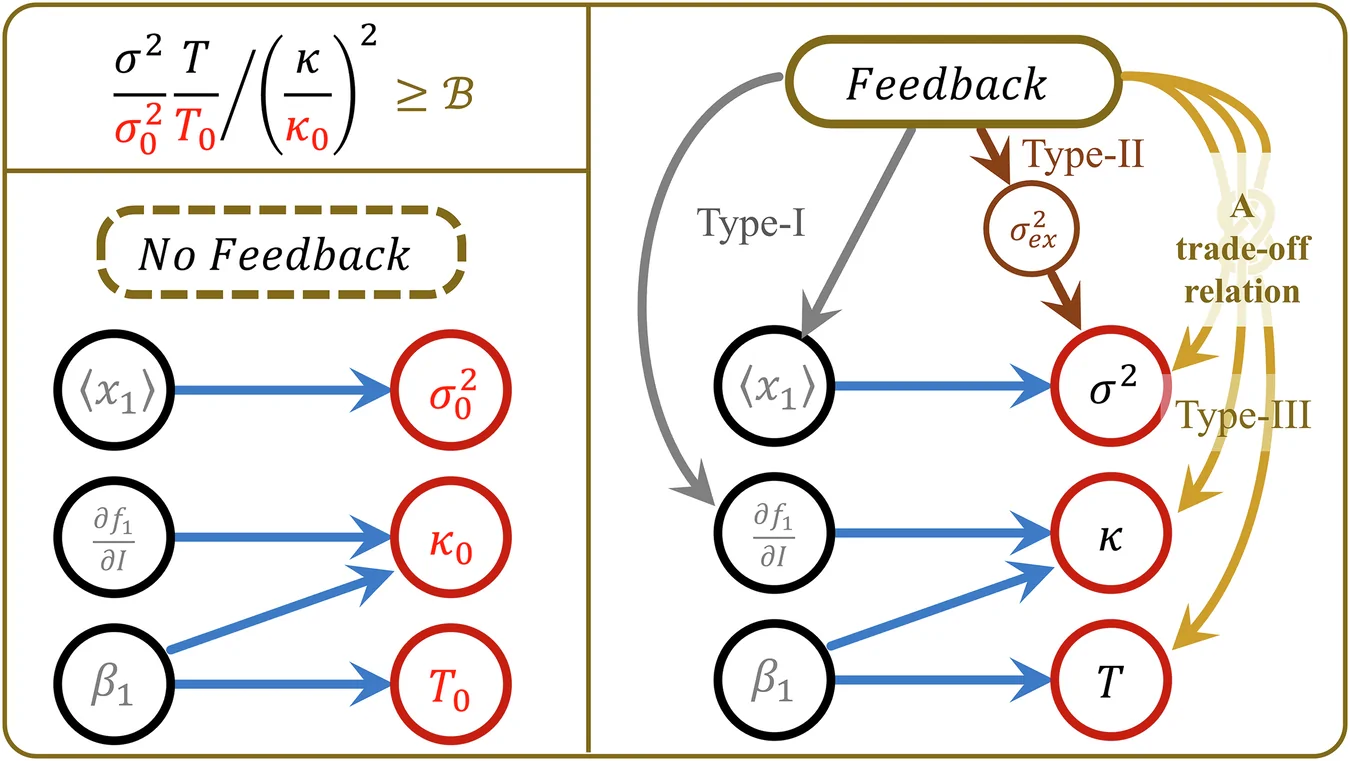
The control effect diagram reflecting three types of effect in feedback control and an interwinding trade-off. The three factors representing the response behavior (fluctuation ${\sigma }^{2}$, sensitivity κ, and timescale T defined in Fig. 1) are denoted in red nodes, which are naturally regulated by three controlled parameters denoted in black nodes and blue arrows in additional to the feedback effect. These regulations also exist in systems without feedback. The type-I effect of feedback (gray arrows) indirectly regulates the response through the controlled parameters, making this effect not unique to feedback. The type-II effect of feedback (brown arrows) introduces addition fluctuation source (brown node), affecting the response fluctuation. The type-III effect (gold arrows) is direct regulations of the three factors by feedback, which is a unique mechanism of feedback control. Our main result (Eq. (8)) indicates that the three type-III effects (the three gold arrows) are mutually constrained, leading to a trade-off relation. Effects of feedback control is characterized by ratio of variables between feedback and feedback-absent condition.

While feedback control generally has multiple effects on the response behavior (e.g. ${\sigma }^{2}$, κ, and T) even in the linear regime, to better disentangle these effects, we propose a “control effect diagram” for a better holistic comprehension (Fig. 6).

We focus on the three factors reflecting the information transmission capacity (red nodes in Fig. 6): the response fluctuation (${\sigma }^{2}$), response sensitivity (κ), and the response timescale (T) which are affected, even in feedback-absent systems, by the “control parameters” (black nodes in Fig. 6): the mean response level ($\left\langle {x}_{1}\right\rangle$), the regulatory strength of the input ($\frac{d{f}_{1}}{{dI}}$), and the decay rate of the response (${\beta }_{1}$), as derived in Eq. (5) (blue arrows in Fig. 6). It is worth emphasizing again that these effects are *not* unique to feedback control.

For feedback control, its effects can generally be categorized into three types: (I) effects on the control parameters $\left\langle {x}_{1}\right\rangle$ and $\frac{d{f}_{1}}{{dI}}$ (gray arrows in Fig. 6); (II) the introduction of additional, independent source of fluctuation (${\sigma }_{{ex}}^{2}$) to the response through the feedback module (brown arrows and brown node in Fig. 6); and (III) direct effects on ${\sigma }^{2}$, κ, and T (gold arrows in Fig. 6).

While all three types of effects affect the response behavior (red nodes in Fig. 6), the type-I indirect effect is relative trivial and not unique to feedback control, i.e., this type of effect can be tuned without the need of a feedback module (as described by Eq. (5)); the type-II effect is pure noise summation, depending on the fluctuation level of the feedback module, and can be arbitrarily small in theory when the molecular number of components in the feedback module is large enough following the Poisson statistics; the type-III effect is the most interesting and unexplored effect unique to feedback control, which is the main focus of our study.

To focus on the type-III effects, we have used the scheme that comparing the feedback-absent condition (Eq. (5)) to eliminate the type-I effects, and omitted the source of fluctuation of the feedback module to avoid the type-II effect; as a result, we can identify a clean trade-off among the three type-III effects (Eq. (8)). In other words, the three gold arrows in Fig. 6 entangle to each other, forming an inevitable trilemma.

## Discussion

Biological regulatory networks must balance multiple competing objectives to transmit signals reliably in the presence of intrinsic noise. While negative feedback is well known to attenuate fluctuations, such isolated observations provide only limited insight into how feedback shapes biological signal transmission as a whole. Here, we show that feedback regulation intrinsically couples three key performance measures—intrinsic fluctuations, response sensitivity, and response speed. The trade-off relation we identify (Eq. (8)), together with the control effect diagram (Fig. 6), provides a unified framework for understanding how feedback simultaneously enhances and constrains regulatory performance.

From a biological perspective, effective signal transmission requires more than noise suppression alone. Regulatory networks must remain sensitive to environmental cues and respond on biologically relevant timescales. Our results demonstrate that these objectives cannot be optimized independently: feedback control cannot arbitrarily suppress intrinsic fluctuations without sacrificing sensitivity or slowing the response. The resulting trade-off thus represents a fundamental limitation on the information transmission capacity of feedback-controlled biological networks.

By explicitly incorporating response timescale, our analysis highlights a factor that is often overlooked in studies of regulatory precision. This finding is consistent with ideas from finite-time thermodynamics, where reducing fluctuations typically incurs a cost in speed or dissipation^49,50^. We further show that nonequilibrium, non-gradient dynamics, common in biological systems, can partially relax this constraint, lowering the minimal cost of noise suppression by at most a factor of two. Importantly, however, the trade-off itself cannot be eliminated, distinguishing it from energy-dependent trade-offs previously identified in biochemical sensing and related processes^51^. Moreover, in stochastic thermodynamics, how dissipation bounds factors, such as fluctuation, response, and timescale, have been revealed over the past decades^15–17,29–31,52–55^, the simultaneous constraint among these factors, however, remains to be explored. Our results imply that simultaneously considering multiple factors could reveal more comprehensive constraints. Notably, the derived bounds (Eqs. (8), (11), (12)) are dynamical bounds instead of thermodynamic bounds. Further theoretical technique is necessitated to characterize the breakdown of detailed balance and thus the free energy dissipation in dynamical systems, such as rewriting the systems in the language of Fokker-Planck equation or chemical master equation^23,38,53,55–58^. Although the degree of non-gradient is tightly connected to the irreversibility of a dynamical system^26,27^, extending the identified bounds to explicitly relate to thermodynamic cost remains an exciting open question.

Our framework also allows the trade-off to be interpreted directly in terms of information transmission—a perspective widely used in analyzing biological systems such as developmental processes^13,32,45,59–63^. Under a small-noise and Gaussian approximation, the mutual information between an input signal and the network response naturally depends on three quantities: intrinsic noise level, response sensitivity, and response timescale. We show that the identified trade-off directly bounds the maximal information gain achievable through feedback control, yielding an upper limit $\Delta {\mathbb{I}}{\mathbb{\le }}{\mathbb{-}}\frac{1}{2}\mathrm{ln}{\mathcal{B}}$, where the factor ${\mathcal{B}}$ is given by the lower bound of the trade-off (Eqs. (8), (11) and (12), for more details, see Supplementary Note 5). This result provides a direct and quantitative link between feedback regulation and information theoretic limits in biological signaling.

Several assumptions underlie our analysis, and we have examined their impact on the robustness of our conclusions. Firstly, if the feedback module per se also suffers from intrinsic fluctuation, i.e., inevitable type-II effect (Fig. 6), the trade-off relation (Eq. (1)) is still valid as the inclusion of any additional sources of fluctuation does not affect the lower bound. Numerical results support this deduction (Supplementary Fig. S4). A potential caveat is that if the intrinsic noise of the feedback module is non-perturbative, carrying fine structure such as colored noise with strong long-term autocorrelation, especially when the whole system is operated beyond the linear regime, the type-II effect could further entangle with both the type-I and the type-III effects. This higher order effect may lead to more intricate phenomena and could potentially alter the optimal trade-off relation. However, these effects are strongly nonlinear and lie beyond the scope of the current study. Nevertheless, a trilemma among fluctuation, sensitivity and speed is still expected.

Secondly, while our analytical results are derived from a linearization based on small-noise limit, the resulting noise level in the numerical simulation is not trivially small (with a typical scale of $\frac{\sqrt{{\sigma }^{2}}}{{\left\langle {x}_{1}\right\rangle }_{\max }-{\left\langle {x}_{1}\right\rangle }_{\min }}\sim 0.1$, Fig. 5(b) and (c), which has the same noise amplitude as in the simulation of Figs. 2 and 3). We also find that if we further increase the fluctuation amplitude by 10-fold in the parameter sampling, the trade-off is still valid (Supplementary Fig. S5), implying the robustness of the derived result in biologically relevant parameter range. Nonetheless, the derivation of the bound (Eq. (8)) is based on linear dynamics. Consequently, our framework does not account for nonlinear effects such as multi-stability, oscillation, adaptation, and others^22–24,57,64^. Therefore, the trade-off relation (Eq. (8)) should not be directly applied to nonlinear behavior without modification. Nevertheless, we still anticipate that a similar trade-off among noise-suppression, sensitivity-enhancement, and speeding-up generally exist (with proper definition for noise, sensitivity and speed) following the spirit of the Data Processing Inequality in information theory. However, it is considerably far more challenging to derive a general mathematical relation valid for nonlinear behavior. And it would require additional mathematical technique, e.g., orbit-averaged quantities for periodic output^22,23,65–68^. This remains an open direction for future studies.

Thirdly, we notice that the definition of sensitivity is usually context-dependent, and our definition of sensitivity, as the derivative of average response with respect to the input (Eq. (3)), differs from those in the studying of adaptation and oscillation^23,24^. This difference is due to the different properties being emphasized, e.g. transient response is emphasized in studying adaptation but steady-state response is emphasized in our study.

Fourthly, while our analysis focuses on continuous dynamical processes modeled by Langevin equations (Eq. (1)), biochemical reactions are sometime, e.g. reactions with small copy numbers, better described by discrete stochastic processes modeled using chemical master equations. In addition, the input signal considered in our model is assumed to be static and change step-like (Fig. 1), which is adopted as a simplification for slow varying quasi-static signal. Some input signals for biochemical networks are, however, varies at the same timescale as, or even faster than, that of the downstream response dynamics. It would be interesting to extend our analysis to such systems.

Finally, our studied framework is composed by a general feedback module and a simplest main pathway (Fig. 1). We notice that this specific setup is a bit unique, but is helpful in constraining our focus on the feedback per se, without distracting toward a too complex main pathway or a more complex control effect diagram (Fig. 6). However, future work is needed to extend our result to a more general framework that the main pathway is more than just one-step cascade. In these extensions, we expect a similar trade-off could be identified, but requiring a more sophisticated comparison (comparing to Eq. (5)), as the control effect diagram will contain more nodes that are affected by the type-I effect of feedback.

More broadly, our study revisits feedback regulation, a foundational concept in systems biology, from the perspective of fundamental limits. While many properties of feedback have been elucidated in networks with simple architectures^5–7,12,28,40,69^, real biological feedback pathways are often highly complex and cannot be adequately described as purely positive or negative. By allowing the feedback module to be complex, our framework moves beyond this dichotomy and reveals constraints that apply generically to feedback-controlled biological networks. These results not only advance our understanding of biological signal transmission but also provide guiding principles for the design of synthetic regulatory circuits operating under intrinsic noise.

## Methods

### Analytical derivation

Given the small noise limit, the Langevin equation (Eq. (1)) is first linearized as $\dot{{\boldsymbol{\delta }}{\boldsymbol{x}}}={\boldsymbol{J}}{\boldsymbol{\delta }}{\boldsymbol{x}}+{J}_{I}\Delta I{{\boldsymbol{e}}}_{{\boldsymbol{1}}}+{\boldsymbol{\eta }}$, where ${\boldsymbol{J}}$ is the Jacobian matrix, which further leads to Eqs. (2) and (3). Based on this linearization, the response fluctuation ${\sigma }^{2}$, sensitivity κ and speed T can be analytically derived for 1D, 2D, and high-dimensional gradient systems (see Supplementary Notes 1 and 2 for details). Combining the expression of these three variables yields the main trade-off relation (Eq. (8)).

### Numerical simulation

Numerical simulations with parameters sampling are performed for both 2D and high-dimensional systems, in which the dynamics described by Eq. (1) is simulated. The regulatory function (${f}_{i}$ in Eq. (1)) is selected as $\frac{{\alpha }_{i}}{1+{e}^{-{\sum }_{j}{R}_{{ij}}\mathrm{ln}\left(1+\frac{{x}_{j}}{{K}_{{ij}}}\right)+{R}_{{Ii}}\mathrm{ln}\left(1+\frac{I}{{K}_{{Ii}}}\right)+{R}_{0i}}}$ mimicking the MWC model dynamics^42–45^, where ${\alpha }_{i}$, ${R}_{{ij}}$, ${K}_{{ij}}$, ${R}_{{Ii}}$, ${K}_{{Ii}}$, and ${R}_{0i}$ are parameters to be sampled. Additional parameters to be sampled included the ${\beta }_{i}$ in Eq. (1), the amplitude of the noise term (${\eta }_{i}$ in Eq. (1)) (see Supplementary Note 4 for detailed parameter sampling scheme).

For the illustrative activator-inhibitor model (Figs. 4 and 5, Eq. (13)), the Hill function is used to model the regulatory function (Eq. (13)). The parameters used in Eq. (13) are: ${\alpha }_{I}={\alpha }_{11}={\alpha }_{12}=\frac{1}{3}$, ${\alpha }_{21}={\alpha }_{22}=\frac{1}{2}$, ${\beta }_{1}={\beta }_{2}=1$, ${K}_{I}={K}_{11}={K}_{12}={K}_{21}={K}_{22}=\frac{1}{2}$, ${n}_{I}=6$, ${n}_{11}=3$, ${n}_{12}=18$, ${n}_{21}=9$, ${n}_{22}=18$.

Numerical simulations are performed using customed MATLAB code.

## Supplementary information

## Data Availability

All relevant data used in this study are available on GitHub at https://github.com/KakitK/FSS_tradeoff.The custom MATLAB code used in this study are available on GitHub at https://github.com/KakitK/FSS_tradeoff.
