# Supplementary material for: A fundamental trade-off among noise suppression, response sensitivity and speed in biological feedback networks
Source: NPJ Syst Biol Appl. 2026 Jun 3;12:132. doi: 10.1038/s41540-026-00755-7 (PMC13538697; doi:10.1038/s41540-026-00755-7)
Supplement: Supplementary file 1 [file 41540_2026_755_MOESM1_ESM.docx]

**Supplementary Information:**

**A fundamental trade-off among noise suppression, response sensitivity and speed in biological feedback networks**

Ka Kit Kong^1,2^, Feng Liu^3*^

^1^ *The State Key Laboratory for Artificial Microstructures and Mesoscopic Physics, School of Physics, Peking University, Beijing, 100871, China*

^2^ *School of Psychological and Cognitive Sciences, Peking University, Beijing, 100871, China*

^3^ *Key Laboratory of Hebei Province for Molecular Biophysics, Institute of Biophysics, School of Health Science & Biomedical Engineering, Hebei University of Technology, Tianjin, 300130, China*

**Contents**

Supplementary Note 1. The fluctuation-sensitivity-speed trade-off in 2D feedback systems	3

Supplementary Note 2. The fluctuation-sensitivity-speed trade-off in gradient systems	4

Supplementary Note 3. The lower bound in 2D systems depends on the degree of non-gradient and the ratio $T/{T_{2}}$	5

Supplementary Note 4. Numerical framework and parameter sampling scheme	7

Supplementary Note 5. Mutual information under small noise and Gaussian approximation	9

Supplementary References	10

Supplementary Figures	12

Supplementary Note 1. The fluctuation-sensitivity-speed trade-off in 2D feedback systems

For 2D systems in which the feedback module is composed of a single node, the dynamic can still generally be described by Eq. (1) as:

$$\dot{x}_{i}=f_{i}\left( \left\{ x_{j} \right\},I \right)-\beta_{i}x_{i}+\eta_{i},$$

(S1)

where $x_{i}$ is the state of node $i$, $i=1, 2$, and ${\partial f_{2}}/{\partial I}=0$ following the scheme in Fig. 1 in the main text. Letting $x_{i}=x_{i,\boldsymbol{s.s.}}+\delta x_{i}$, Eq. (S1) can be linearized as:

$$\dot{\boldsymbol{\delta x}}=\boldsymbol{J\delta x}+J_{I}\Delta I\boldsymbol{e}_{\boldsymbol{1}}+\boldsymbol{\eta},$$

(S2)

where $J_{ij}={\partial\left( f_{i}-\beta_{i}x_{i} \right)}/{\partial x_{j}}$ is the Jacobian, $J_{I}={\partial f_{1}}/{\partial I}$. Solving Eq. (S2) in the zero noise limit approximately yields the sensitivity $\kappa=\frac{d\delta x_{1}}{dI}=-\boldsymbol{e}_{\boldsymbol{1}}^{\boldsymbol{T}}\boldsymbol{J}^{\boldsymbol{-1}}\boldsymbol{e}_{\boldsymbol{1}}J_{I}=-\frac{J_{22}}{\Delta}J_{I}$, where $\Delta=\det\left( J \right)>0$.

The covariance matrix $\boldsymbol{\Sigma}_{x}=\left\langle\boldsymbol{\delta x\delta}\boldsymbol{x}^{\boldsymbol{T}} \right\rangle$ follows the Lyapunov equation[1,2]:

$$\boldsymbol{J}\boldsymbol{\Sigma}_{\boldsymbol{x}}\boldsymbol{+}\boldsymbol{\Sigma}_{\boldsymbol{x}}\boldsymbol{J}^{\boldsymbol{T}}\boldsymbol{+D}=\boldsymbol{0},$$

(S3)

where $\boldsymbol{D=}\left\langle\boldsymbol{\eta}\boldsymbol{\eta}^{\boldsymbol{T}} \right\rangle$ is the diffusion matrix. In our model, we assume that the feedback module does not introduce additional noise sources, which means $D_{11}=D_{1}\neq0$, and $D_{ij}=0, \forall\left( i,j \right)\neq\left( 1,1 \right)$. Solving Eq. (S3) under this condition yields the response fluctuation $\sigma^{2}=\left\langle\delta x_{1}^{2} \right\rangle=\boldsymbol{e}_{\boldsymbol{1}}^{\boldsymbol{T}}\boldsymbol{\Sigma}_{\boldsymbol{x}}\boldsymbol{e}_{\boldsymbol{1}}=-\frac{D_{1}(J_{22}^{2}+\Delta)}{2\Delta\tau}$, where $\tau=\mathrm{tr}\left( \boldsymbol{J} \right)<0$. Note that although the form of $D_{1}$ applied in the main text represents the simplest intrinsic Poisson noise, a different $D_{1}$ corresponding to a different noise does not change the result, as its effect is canceled out in the normalization scheme (i.e., $\frac{\sigma^{2}}{\sigma_{0}^{2}}$) and thus the form of $D_{1}$ is not involved in the following derivation.

Combining the response sensitivity and fluctuation, with that of the no-feedback condition: $\kappa_{0}=\frac{J_{I}}{\beta_{1}},\sigma_{0}^{2}=\frac{D_{1}}{2\beta_{1}}$ (Eq. (5)), yields the trade-off relation (Eq. (8) in the main text):

$${\left( \frac{\kappa}{\kappa_{0}} \right)^{2}}/\left( \frac{\sigma^{2}}{\sigma_{0}^{2}} \right)=-\frac{J_{22}^{2}\beta_{1}^{2}}{\Delta^{2}}\frac{\Delta\tau}{\beta_{1}\left( J_{22}^{2}+\Delta\right)}=-\beta_{1}\frac{\tau}{\Delta}\frac{J_{22}^{2}}{\left( J_{22}^{2}+\Delta\right)}<-\beta_{1}\frac{\tau}{\Delta}\leq2\frac{T}{T_{0}},$$

(S4)

where the first inequality is $\frac{J_{22}^{2}}{\left( J_{22}^{2}+\Delta\right)}<1$ since $\Delta>0$. The second inequality is $-\beta_{1}\frac{\tau}{\Delta}\leq2\frac{T}{T_{0}}$, where we have used $T_{0}=1/{\beta_{1}}$ and $-\tau/\Delta\leq2T$. The last relation is derived as follows: if the two eigenvalues of $\boldsymbol{J}$ are real ($\lambda_{1,2}\mathbb{\in R}$), we have $-\frac{\tau}{\Delta}=-\frac{\lambda_{1}+\lambda_{2}}{\lambda_{1}\lambda_{2}}=\frac{\frac{1}{T_{1}}+\frac{1}{T_{2}}}{\frac{1}{T_{1}T_{2}}}=T_{1}+T_{2}\leq2T$, where $T_{i}=-1/{\lambda_{i}}$ and the timescale $T$ is defined as the slowest timescale of the dynamic $T=\max\left\{ T_{i} \right\}$. If the two eigenvalues of $\boldsymbol{J}$ are complex ($\lambda_{1,2}\mathbb{\in C}$), let $\lambda_{1,2}=\lambda_{r}\pm i\lambda_{c}$, we have $-\frac{\tau}{\Delta}=2\frac{-\lambda_{r}}{\lambda_{r}^{2}+\lambda_{c}^{2}}<2\frac{-1}{\lambda_{r}}=2T$, where the relaxation timescale of the dynamic is $T=\frac{-1}{\lambda_{r}}$. Eq. (S4) gives the trade-off relation Eq. (8).

It is worth noting that combining $\frac{\sigma^{2}}{\sigma_{0}^{2}}=-\frac{\beta_{1}(J_{22}^{2}+\Delta)}{\Delta\tau}$ and $\frac{\kappa}{\kappa_{0}}=-\frac{J_{22}}{\Delta}\beta_{1}$ gives:

$${\frac{\sigma^{2}}{\sigma_{0}^{2}}}/\left| \frac{\kappa}{\kappa_{0}} \right|=\frac{J_{22}^{2}+\Delta}{\left| J_{22}\tau\right|}\geq\frac{2\left| J_{22} \right|\sqrt{\Delta}}{\left| J_{22} \right|\left| \tau\right|}=2\sqrt{\frac{\Delta}{\tau^{2}}}\geq2\frac{\sqrt{T/{T_{\min}}}}{1+T/{T_{\min}}}.$$

(S5)

The first inequality is $J_{22}^{2}+\Delta\geq2\sqrt{J_{22}^{2}\Delta}$ because $J_{22}^{2}\geq0$, $\Delta>0$. The last inequality is derived as follows: if the two eigenvalues are real, we have $2\sqrt{\frac{\Delta}{\tau^{2}}}=2\sqrt{\frac{\lambda_{1}\lambda_{2}}{\left( \lambda_{1}+\lambda_{2} \right)^{2}}}=2\sqrt{\frac{T_{1}T_{2}}{\left( T_{1}+T_{2} \right)^{2}}}=2\sqrt{\frac{T/T_{\min}}{\left( 1+T/T_{\min} \right)^{2}}}$; if the two eigenvalues are complex ($\lambda_{1,2}=\lambda_{r}\pm i\lambda_{c}$), $2\sqrt{\frac{\Delta}{\tau^{2}}}=2\sqrt{\frac{\lambda_{r}^{2}+\lambda_{c}^{2}}{4\lambda_{r}^{2}}}=\sqrt{1+\frac{\lambda_{c}^{2}}{\lambda_{r}^{2}}}>1=2\frac{\sqrt{T/T_{\min}}}{1+T/T_{\min}}$, as the timescale is defined based on the real part of the eigenvalue, thus $T=T_{\min}={-1}/{\lambda_{r}}$, leading to $2\frac{\sqrt{T/T_{\min}}}{1+T/T_{\min}}=1$ in this condition. Eq. (S5) indicates that there is no non-zero lower bound for the ratio of noise and sensitivity, as long as the dynamic exhibits a timescale separation ($T/{T_{\min}}\gg1$) (Fig. S1, Numerical details in Supplementary Note 4). This is consistent with the trade-off (Eq. (S4)), both of which suggest that infinitely slow dynamics can achieve arbitrary small noise with given sensitivity. It is worth noting that the apparent lower bound in Fig. S1 is due to the limited parameter sampling range (${10}^{0}\leq T/{T_{\min}}\leq{10}^{2}$), the trend of an unlimited lower bound can be seen from the dots’ color.

Supplementary Note 2. The fluctuation-sensitivity-speed trade-off in gradient systems

For high dimensional gradient systems, the linearized equation has the same form as Eq. (S2), with $i=1,2,\ldots$ and an additional constraint that $\boldsymbol{J=}\boldsymbol{J}^{\boldsymbol{T}}$, and thus all eigenvalues of $\boldsymbol{J}$ are real. For clarity, we denote the eigenvalue of $\boldsymbol{J}$ as $-\tilde{\lambda}_{i}$, therefore $\tilde{\lambda}_{i}>0$. Based on the eigen decomposition of $\boldsymbol{J=-P}\boldsymbol{\Lambda}\boldsymbol{P}^{\boldsymbol{T}}$, where $\boldsymbol{P}^{\boldsymbol{T}}\boldsymbol{=}\boldsymbol{P}^{\boldsymbol{-1}}$, and $\boldsymbol{\Lambda}$ is a real diagonal matrix with elements $\left\{ \tilde{\lambda}_{i} \right\}$, Eq. (S2) can be rewritten as:

$$\dot{y_{i}}=-\tilde{\lambda}_{i}y_{i}+v_{i}J_{I}\Delta I+v_{i}\eta_{1},$$

(S6)

where $\boldsymbol{Y}=\left( y_{1},y_{2},\ldots\right)^{T}=\boldsymbol{P}^{\boldsymbol{T}}\boldsymbol{\delta X}$, $\boldsymbol{v}=\left( v_{1},v_{2},\ldots\right)^{T}=\boldsymbol{P}^{\boldsymbol{T}}\boldsymbol{e}_{\boldsymbol{1}}$. The covariance of $y$ is $\left\langle y_{i}y_{j} \right\rangle=\frac{v_{i}v_{j}}{\tilde{\lambda}_{i}+\tilde{\lambda}_{j}}D_{1}$, thus the response fluctuation is given by $\sigma^{2}=\boldsymbol{e}_{\boldsymbol{1}}^{\boldsymbol{T}}\boldsymbol{\Sigma}_{\boldsymbol{x}}\boldsymbol{e}_{\boldsymbol{1}}=\left( \boldsymbol{P}^{\boldsymbol{T}}\boldsymbol{e}_{\boldsymbol{1}} \right)^{\boldsymbol{T}}\boldsymbol{\Sigma}_{\boldsymbol{y}}\left( \boldsymbol{P}^{\boldsymbol{T}}\boldsymbol{e}_{\boldsymbol{1}} \right)=\boldsymbol{v}^{\boldsymbol{T}}\boldsymbol{\Sigma}_{\boldsymbol{y}}\boldsymbol{v}=\sum_{i} \sum_{j} \frac{v_{i}^{2}v_{j}^{2}}{\tilde{\lambda}_{i}+\tilde{\lambda}_{j}}D_{1}\geq\sum_{i} \sum_{j} \frac{v_{i}^{2}v_{j}^{2}}{2\max\left\{ \tilde{\lambda}_{i},\tilde{\lambda}_{j} \right\}}D_{1}$.

The response sensitivity is derived as follows: Eq. (S6) gives $\frac{dy_{i}}{dI}=\frac{v_{i}}{\tilde{\lambda}_{i}}J_{I}$ under the zero noise condition, the sensitivity is $\kappa=\frac{dx_{1}}{dI}=\frac{d}{dI}\left( \boldsymbol{e}_{\boldsymbol{1}}^{\boldsymbol{T}}\boldsymbol{PY} \right)=\frac{d}{dI}\left( \boldsymbol{v}^{\boldsymbol{T}}\boldsymbol{Y} \right)=\sum_{i} \frac{v_{i}^{2}}{\tilde{\lambda}_{i}}J_{I}$, leading to $\kappa^{2}=\sum_{i} \sum_{j} \frac{v_{i}^{2}v_{j}^{2}}{\tilde{\lambda}_{i}\tilde{\lambda}_{j}}J_{I}^{2}\leq\sum_{i} \sum_{j} \frac{1}{\tilde{\lambda}_{\min}}\frac{v_{i}^{2}v_{j}^{2}}{\max\left\{ \tilde{\lambda}_{i},\tilde{\lambda}_{j} \right\}}J_{I}^{2}$.

Combining the closed-form of $\sigma^{2}$, $\kappa^{2}$, $T=1/{\tilde{\lambda}_{\min}}$, and the normalization factors $T_{0}=1/{\beta_{1}}$, $\sigma_{0}^{2}={D_{1}}/{2\beta_{1}}$, and $\kappa_{0}={{J_{I}}/\beta}_{1}$ (Eq. (5)), yields the trade-off relation: ${\frac{\sigma^{2}}{\sigma_{0}^{2}}\frac{T}{T_{0}}}/{\left( \frac{\kappa}{\kappa_{0}} \right)^{2}}\geq1$ (Eq. (9) in the main text, Fig. S2).

Supplementary Note 3. The lower bound in 2D systems depends on the degree of non-gradient and the ratio $\boldsymbol{T}/{\boldsymbol{T}_{\boldsymbol{2}}}$

To derive the lower bound depending on the degree of non-gradient and the ratio $T/{T_{2}}$, we first introduce the notations used in the derivation. First, the Jacobian of the linearized equation Eq. (S2) is re-written as: $\boldsymbol{J=}\boldsymbol{J}^{\mathbf{s}}\boldsymbol{+}\boldsymbol{J}^{\mathbf{as}}$, where $\boldsymbol{J}^{\mathbf{s}}=\left( \boldsymbol{J+}\boldsymbol{J}^{\boldsymbol{T}} \right)/2=\left( \begin{matrix} J_{1} & J_{\times} \\ J_{\times} & J_{2} \end{matrix} \right)$ is symmetric, and $\boldsymbol{J}^{\mathbf{as}}=\left( \boldsymbol{J-}\boldsymbol{J}^{\boldsymbol{T}} \right)/2=\left( \begin{matrix} 0 & \epsilon\\ -\epsilon& 0 \end{matrix} \right)$ is antisymmetric. The determinant of $\boldsymbol{J}$ is $\Delta=\det\left( \boldsymbol{J} \right)=J_{1}J_{2}-J_{\times}^{2}+\epsilon^{2}=\Delta_{0}+\epsilon^{2}$, where $\Delta_{0}=J_{1}J_{2}-J_{\times}^{2}$ is the determinant of $\boldsymbol{J}^{\mathbf{s}}$**.** The trace of $\boldsymbol{J}$ equals that of $\boldsymbol{J}^{\mathbf{s}}$, denoted as $\tau=J_{1}+J_{2}$. The eigenvalues of $\boldsymbol{J}$ is $\lambda_{1}=\frac{1}{2}\left( \tau+\sqrt{\delta} \right)$ and $\lambda_{2}=\frac{1}{2}\left( \tau-\sqrt{\delta} \right)$, where $\delta=\tau^{2}-4\Delta$. Since the fixed point is stable, a constraint is $\mathrm{Re}\left( \lambda_{1,2} \right)<0$ and $\tau<0$. The eigenvalues of $\boldsymbol{J}^{\mathbf{s}}$ is $\lambda_{s1}=\frac{1}{2}(\tau+\sqrt{\delta+4\epsilon^{2}})$ and $\lambda_{s2}=\frac{1}{2}(\tau-\sqrt{\delta+4\epsilon^{2}})$. Since $\tau<0$ and $\boldsymbol{J}^{\mathbf{s}}$ is symmetric, we have $\lambda_{s1,s2}\mathbb{\in R}$, which requires $\delta+4\epsilon^{2}\geq0$, and $\left| \lambda_{s2} \right|\geq\left| \lambda_{s1} \right|$. The degree of non-gradient is $\chi=\frac{\mathrm{im}\left( \lambda_{1}\left( \boldsymbol{J}^{\mathbf{a}\mathbf{s}} \right) \right)}{\mathrm{im}\left( \lambda_{1}\left( \boldsymbol{J}^{\mathbf{a}\mathbf{s}} \right) \right)-\lambda_{1}\left( \boldsymbol{J}^{\mathbf{s}} \right)}=\frac{\epsilon}{\epsilon-\lambda_{s1}}$. Since $J_{\times}\mathbb{\in R}$ and $J_{\times}^{2}=\tau J_{2}-J_{2}^{2}-\Delta_{0}$, another constraint is $\lambda_{s2}\leq J_{2}\leq\lambda_{s1}$. It is worth noting that it is possible $\lambda_{s1}\geq0$. With these notations, we next derive the lower bound $\mathcal{B}_{G}\left( \chi\right)$ started with Eq. (S4):

$${\frac{\sigma^{2}}{\sigma_{0}^{2}}\frac{T}{T_{0}}}/{\left( \frac{\kappa}{\kappa_{0}} \right)^{2}}=-T\frac{\Delta}{\tau}\left( 1+\frac{\Delta}{J_{2}^{2}} \right).$$

(S7)

We analyze Eq. (S7) based on whether $\delta\geq0$or $\delta<0$.

If $\delta\geq0$, $\lambda_{1,2}\mathbb{\in R}$, and $T=-1/{\lambda_{1}}$, Eq. (S7) becomes:

$${\frac{\sigma^{2}}{\sigma_{0}^{2}}\frac{T}{T_{0}}}/{\left( \frac{\kappa}{\kappa_{0}} \right)^{2}}=\frac{\lambda_{2}}{\lambda_{1}+\lambda_{2}}\left( 1+\frac{\Delta}{J_{2}^{2}} \right)\geq\frac{\lambda_{2}}{\lambda_{1}+\lambda_{2}}\left( 1+\frac{\Delta}{\lambda_{s2}^{2}} \right)=\frac{\lambda_{2}}{\lambda_{1}+\lambda_{2}}\left[ 1+\frac{\Delta}{\left( \lambda_{s1}-\sqrt{\delta+4\epsilon^{2}} \right)^{2}} \right]=\frac{1}{2}\left( 1-\frac{\sqrt{z-4\epsilon^{2}}}{2\lambda_{s1}-\sqrt{z}} \right)\left[ 1+\frac{1}{4}\frac{\left( 2\lambda_{s1}-\sqrt{z} \right)^{2}-z+4\epsilon^{2}}{\left( \lambda_{s1}-\sqrt{z} \right)^{2}} \right]\triangleq f\left( z,\epsilon,\lambda_{s1} \right),$$

(S8)

where we have used $J_{2}^{2}\leq\lambda_{s2}^{2}$ in the first inequality, $z=\delta+4\epsilon^{2}$, $f\left( z,\epsilon,\lambda_{s1} \right)=\frac{1}{2}\left( 1-\frac{\sqrt{z-4\epsilon^{2}}}{2\lambda_{s1}-\sqrt{z}} \right)\left[ 1+\frac{1}{4}\frac{\left( 2\lambda_{s1}-\sqrt{z} \right)^{2}-z+4\epsilon^{2}}{\left( \lambda_{s1}-\sqrt{z} \right)^{2}} \right]$. Under the constraint $\delta\geq0, z\geq4\epsilon^{2}$, minimizing $f\left( z,\epsilon,\lambda_{s1} \right)$ with respect to $z$ gives:

$${\frac{\sigma^{2}}{\sigma_{0}^{2}}\frac{T}{T_{0}}}/{\left( \frac{\kappa}{\kappa_{0}} \right)^{2}}\geq f\left( z=4\epsilon^{2},\epsilon,\lambda_{s1} \right)=\frac{1}{2}\left[ 1+\left( \frac{1}{1+\chi} \right)^{2} \right].$$

(S9)

If $\delta<0$, $\lambda_{1,2}\mathbb{\in C}$, and $T=-2/\tau$, Eq. (S7) becomes:

$${\frac{\sigma^{2}}{\sigma_{0}^{2}}\frac{T}{T_{0}}}/{\left( \frac{\kappa}{\kappa_{0}} \right)^{2}}=2\frac{\Delta}{\tau^{2}}\left( 1+\frac{\Delta}{J_{2}^{2}} \right)\geq2\frac{\Delta}{\tau^{2}}\left( 1+\frac{\Delta}{\lambda_{s2}^{2}} \right)\triangleq g\left( z,\epsilon,\lambda_{s1} \right),$$

where we have used $J_{s}^{2}\leq\lambda_{s2}^{2}$, $g\left( z,\epsilon,\lambda_{s1} \right)=\frac{1}{2}\left[ 1-\frac{z-4\epsilon^{2}}{\left( 2\lambda_{s1}-\sqrt{z} \right)^{2}} \right]\left[ 1+\frac{1}{4}\frac{\left( 2\lambda_{s1}-\sqrt{z} \right)^{2}-z+4\epsilon^{2}}{\left( \lambda_{s1}-\sqrt{z} \right)^{2}} \right]$. Under the condition $\delta<0, 0\leq z<4\epsilon^{2}$ (since $\lambda_{s1,s2}\mathbb{\in R}$), minimizing $g\left( z,\epsilon,\lambda_{s1} \right)$ with respect to $z$ gives:

$${\frac{\sigma^{2}}{\sigma_{0}^{2}}\frac{T}{T_{0}}}/{\left( \frac{\kappa}{\kappa_{0}} \right)^{2}}\geq g\left( z=4\epsilon^{2},\epsilon,\lambda_{s1} \right)=\frac{1}{2}\left[ 1+\left( \frac{1}{1+\chi} \right)^{2} \right].$$

(S10)

Combining Eqs. (S9) and (S10) yields the lower bound in 2D system:

$${\frac{\sigma^{2}}{\sigma_{0}^{2}}\frac{T}{T_{0}}}/{\left( \frac{\kappa}{\kappa_{0}} \right)^{2}}\geq\mathcal{B}_{G}\left( \chi\right)=\frac{1}{2}\left[ 1+\left( \frac{1}{1+\chi} \right)^{2} \right].$$

Regarding the lower bound depending on $T/{T_{2}}$, when $\delta\geq0$, Eq. (S8) gives:

$${\frac{\sigma^{2}}{\sigma_{0}^{2}}\frac{T}{T_{0}}}/{\left( \frac{\kappa}{\kappa_{0}} \right)^{2}}\geq\frac{\lambda_{2}}{\lambda_{1}+\lambda_{2}}\left( 1+\frac{\Delta}{\lambda_{s2}^{2}} \right)>\frac{\lambda_{2}}{\lambda_{1}+\lambda_{2}}=\frac{T/{T_{2}}}{1+T/{T_{2}}}.$$

(S11)

If $\delta<0$, from the definition of timescale, we have $T/{T_{2}}=1$, and the lower bound is $1/2$, which is consistent with Eq. (S11).

Supplementary Note 4. Numerical framework and parameter sampling scheme

Numerically, we model the dynamic of complex regulatory networks using the Monod-Wyman-Changeux (MWC) model [3–6], in which the concentration (or activity) of the node $i$ ($x_{i}$) follows:

$$\frac{dx_{i}}{dt}=\alpha_{i}f\left( z_{i} \right)-\beta_{i}x_{i}+\eta_{i}.$$

(S12)

where $x_{i}$ is normalized such that $x_{i,s.s.}\in\left[ 0,1 \right]$ by letting $\alpha_{i}=\beta_{i}$, and the intrinsic fluctuation $\eta_{i}$ follows $\left\langle\eta_{i}\left( t \right)\eta_{j}\left( t^{'} \right) \right\rangle=D_{i}\delta\left( t-t^{'} \right)\delta_{ij}$. Here $D_{i}=\frac{1}{C_{i}}\left( \alpha_{i}f\left( z_{i} \right)+\beta_{i}x_{i} \right)$ is the renormalized fluctuation amplitude, $C_{i}$ represents the maximal number of molecules, i.e., the actual molecule number is $x_{i}C_{i}$, $f\left( z \right)=\frac{1}{1+e^{-z}}$ is the regulatory function, $z_{i}=\sum_{j} R_{ij}\ln\left( 1+\frac{x_{j}}{K_{ij}} \right)+R_{Ii}\ln\left( 1+\frac{I}{K_{Ii}} \right)+R_{0i}$ represent the regulatory network, $R_{ij}>0$ indicates a positive regulation and vice versa. Under the assumption that the feedback module does not introduce additional noise, we assume $D_{i}=0, \forall i\neq1$, by assuming the absolute copy number of the feedback module species is infinite $C_{i}\to\infty, \forall i\neq1$. The valid of $C_{1}$ is chosen as $100$.

For the parameter sampling, we first randomly choose $\alpha_{i}=\beta_{i}$ following a log-uniform distribution from ${10}^{-1}$ to ${10}^{1}$; $K_{ij}$, $K_{Ii}$, and $R_{I1}$ also follow a log-uniform distribution from ${10}^{-1}$ to ${10}^{1}$, $R_{Ii}=0,\forall i\neq1$; auxiliary parameters indicating the steady-state $x_{i,s.s.}$ are then explicitly sampled following a uniform distribution from 0.01 to 0.99. Next, we sample a set of auxiliary parameters indicating the negative real part and imaginary part of the eigenvalues of the Jacobian: $\left\{ \tilde{\lambda}_{i} \right\}$, the minimum of $\tilde{\lambda}_{i}$ is first sampled following a log-uniform distribution from ${10}^{-1}$ to ${10}^{1}$, the second minimum of $\tilde{\lambda}_{i}$ is then sampled following a log-uniform distribution from the minimum sampled before to ${10}^{1}$, the remaining $\tilde{\lambda}_{i}$ is sampled following a log-uniform distribution from the second minimum to ${10}^{1}$. With the sampled set of parameters $\left\{ \tilde{\lambda}_{i} \right\}$, a random fraction is chosen to be the real part of the eigenvalues denoting as $\left\{ \tilde{\lambda}_{ri} \right\}$, the remaining fraction denoting as $\left\{ \tilde{\lambda}_{ci} \right\}$ is the imaginary part. The Jacobian is constructed following $\boldsymbol{J=PB}\boldsymbol{P}^{\boldsymbol{-1}}$, where $\boldsymbol{B}$ is a blocky diagonal matrix with block $\left[ -\tilde{\lambda}_{ri} \right]$ or $\left[ \begin{matrix} -\tilde{\lambda}_{ri} & \tilde{\lambda}_{ci} \\ -\tilde{\lambda}_{ci} & -\tilde{\lambda}_{ri} \end{matrix} \right]$, each $\tilde{\lambda}_{i}$ appears in one and only one block of $\boldsymbol{B}$, $\boldsymbol{P}$ is a random matrix with elements generated first following a Gaussian distribution, each column of $\boldsymbol{P}$ is then normalized separately and assigned a random length following a uniform distribution from 0 to 1. With these sampled parameters and auxiliary parameters, $R_{ij}$ and $R_{0i}$ can be determined accordingly. The input $I$ follows a uniform distribution from 0 to 1.

For numerical results of local gradient systems (Fig. S2), all eigenvalues are set to be real, and the random matrix $\boldsymbol{P}$ is set to be orthogonal such that the Jacobian $\boldsymbol{J}$ is symmetric. For numerical results in which the feedback module does introduce additional fluctuation (Fig. S4), we set $C_{i}=100, \forall i$. For numerical results with enhanced fluctuation amplitude (Fig. S5), we set $C_{1}=10$, and $C_{i}\to+\infty, \forall i\neq1$.

For all numerical results, we perform a simulation with the same set of parameters at the same time, but without any noise, to estimate the deterministic response $x_{\det,1}$. Under the small noise limit, $x_{\det,1}=\left\langle x_{1} \right\rangle$, therefore we use the deterministic response to estimate the response sensitivity $\kappa\approx\frac{dx_{1,det}}{dI}\approx\frac{x_{1,\det}\left( I_{0} \right)-x_{1,\det}\left( I_{0}+\Delta I \right)}{\Delta I}$ to avoid numerical error, in which $\Delta I={10}^{-10}$. This procedure is applied because the direct estimation of $\left\langle x_{1} \right\rangle$ from stochastic simulations is extremely noisy, involving inevitable numerical errors. To ensure the validity of this estimation, we exclude results in which the difference between $x_{i,det}$ and $\left\langle x_{i} \right\rangle$ is larger than $0.001$. Moreover, since our parameter sampling scheme has a pre-defined steady-state $x_{i,s.s.}$, we also exclude results in which the difference between $x_{i,det}$ and $x_{i,s.s.}$ or the difference between $\left\langle x_{i} \right\rangle$ and $x_{i,s.s.}$ is greater than $0.001$. To ensure precision in estimating $\kappa$, a second estimation of $\kappa$ is evaluated with $\Delta I=-{10}^{-10}$. If these two estimations differ more than $50\%$, the results are excluded.

Supplementary Note 5. Mutual information under small noise and Gaussian approximation

In this part, we use the mutual information (between the input $I$ and the response $R$, denoted as $\mathbb{I}\left( R;I \right)$) to quantify the information transmission of the feedback network in Fig. 1. By definition,

$$\mathbb{I}\left( R;I \right)=S\left( R \right)-S\left( R|I \right),$$

(S13)

where $S\left( \ldots\right)$ is the differential entropy. The response $R$ is considered to be a temporal averaging of the fluctuating output of the network $x_{1}$ through a finite time windows $T_{read}$, i.e., $R=\frac{1}{T_{read}}\int_{T_{read}} x_{1}\left( t \right)dt$.

Assuming the distribution of response $R$ under given input is a Gaussian, we have $S\left( R|I \right)=\left\langle\frac{1}{2}\ln2\pi e+\frac{1}{2}\ln\sigma_{R|I}^{2} \right\rangle_{I}$, where $\sigma_{R|I}^{2}$ is the variance of $R$ under given input $I$, $\left\langle\ldots\right\rangle_{I}$ indicate average over the distribution of input $I$. Since the response $R$ is a temporal averaging of the $x_{1}$, we have $\sigma_{R|I}^{2}=\sigma^{2}\frac{T_{corr}}{T_{read}}$, where $\sigma^{2}$ is the variance of $x_{1}$, $T_{corr}$ is the autocorrelation time of the fluctuating $x_{1}$, which shares the scale with the intrinsic dynamic timescale $T_{corr}\sim T$. Therefore, approximately we have $S\left( R|I \right)\approx\frac{1}{2}\ln\frac{2\pi e}{T_{read}}+{\frac{1}{2}\left\langle\ln\sigma^{2}T \right\rangle}_{I}$.

The $S\left( R \right)$ in Eq. (S13) relies on the marginal distribution of $R$ which can be estimated by assuming the noise $\sigma_{R|I}^{2}$ is small enough. Under this small noise approximation, we have

$$P\left( R \right)=\int P\left( R|I \right)P(I)dI=\int\frac{1}{\sqrt{2\pi\sigma_{R|I}^{2}}}e^{-\frac{\left[ R-\mu\left( I \right) \right]^{2}}{2\sigma_{R|I}^{2}}}P\left( I \right)dI\overset{\sigma_{R|I}^{2}\approx0}{\approx}\left| \frac{P\left( I \right)}{{d\mu}/{dI}} \right|_{\mu\left( I \right)=R}=\left| \frac{P\left( I \right)}{\kappa\left( I \right)} \right|_{\mu\left( I \right)=R},$$

where $\mu\left( I \right)=\left\langle R \right\rangle_{I}=\left\langle x_{1} \right\rangle_{I}$, $\kappa=\frac{d\mu}{dI}$ is the sensitivity we discuss throughout the paper. Therefore, the entropy of response $R$ is

$$S\left( R \right)=-\int P\left( R \right)\ln P\left( R \right)dR=-\int P\left( I \right)\ln P\left( I \right)dI+\frac{1}{2}\int P\left( I \right)\ln\kappa^{2}\left( I \right)dI=S\left( I \right)+\frac{1}{2}\left\langle\ln\kappa^{2} \right\rangle_{I},$$

where $S\left( I \right)=-\int P\left( I \right)\ln P\left( I \right)dI$ is the differential entropy of the input distribution. Mathematically, a monotonic $\mu\left( I \right)$ is required, which is equivalent to require small inference noise $\sigma_{I|R}^{2}\approx0$ or $\frac{\sigma_{R|I}^{2}}{\left( {d\mu}/{dI} \right)^{2}}\approx0$.

Plugging these results into Eq. (S13) gives the mutual information as:

$$\mathbb{I=}C+\frac{1}{2}\left\langle\ln\frac{\kappa^{2}}{\sigma^{2}T} \right\rangle_{I},$$

(S14)

where $C=S\left( I \right)-\frac{1}{2}\ln\frac{2\pi e}{T_{read}}$ is a constant. The derivation of the expression does not assume the existence of the feedback module. In other word, Eq. (S14) also valid in the feedback-absent condition, the only different is the value of $\kappa$, $\sigma^{2}$ and $T$, i.e., the mutual information in the feedback-absent condition is:

$$\mathbb{I}_{0}=C+\frac{1}{2}\left\langle\ln\frac{\kappa_{0}^{2}}{\sigma_{0}^{2}T_{0}} \right\rangle_{I}.$$

Therefore, the information increment due to the feedback is $\Delta\mathbb{I=I-}\mathbb{I}_{0}=-\frac{1}{2}\left\langle\ln{\frac{\sigma^{2}}{\sigma_{0}^{2}}\frac{T}{T_{0}}}/{\left( \frac{\kappa}{\kappa_{0}} \right)^{2}} \right\rangle_{I}\leq-\frac{1}{2}\ln\mathcal{B}$, where the main result of this paper ${\frac{\sigma^{2}}{\sigma_{0}^{2}}\frac{T}{T_{0}}}/{\left( \frac{\kappa}{\kappa_{0}} \right)^{2}}\mathcal{\geq B}$ is used.

Supplementary References

1.	Paulsson J. Models of stochastic gene expression. Phys Life Rev. 2005;2: 157–175. doi:10.1016/j.plrev.2005.03.003

2.	Paulsson J. Summing up the noise in gene networks. Nature. 2004;427: 415–418. doi:10.1038/nature02257

3.	Monod J, Wyman J, Changeux J-P. On the nature of allosteric transitions: A plausible model. J Mol Biol. 1965;12: 88–118. doi:10.1016/S0022-2836(65)80285-6

4.	Grah R, Zoller B, Tkačik G. Nonequilibrium models of optimal enhancer function. Proc Natl Acad Sci. 2020; 202006731. doi:10.1073/pnas.2006731117

5.	Walczak AM, Tkačik G, Bialek W. Optimizing information flow in small genetic networks. II. Feed-forward interactions. Phys Rev E. 2010;81: 041905. doi:10.1103/PhysRevE.81.041905

6.	Sokolowski TR, Gregor T, Bialek W, Tkačik G. Deriving a genetic regulatory network from an optimization principle. Proc Natl Acad Sci. 2025;122: e2402925121. doi:10.1073/pnas.2402925121

Supplementary Figures

Supplementary Figure 1. The fluctuation and response sensitivity in 2D and 10D dynamics. (a, b) Numerical results show that there is no non-zero lower bound for the $\sigma^{2}-\kappa$ relation in 2D (a) and 10D (b) dynamics. The apparent lower bound is due to the limited sampling range of the parameter $T/{T_{\min}}$. As indicated by the dots’ color, the apparent lower bound of $\sigma^{2}$ keeps decreasing as the range of $T/{T_{\min}}$ increases.

Supplementary Figure 2. The fluctuation-sensitivity-timescale trade-off in 2D and 10D gradient systems, compared to Fig. 2 in the main text. (a, b) Numerical results of 2D (a) and 10D (b) dynamics show that the trade-off for gradient systems is ${\frac{\sigma^{2}}{\sigma_{0}^{2}}\frac{T}{T_{0}}}/{\left( \frac{\kappa}{\kappa_{0}} \right)^{2}}\geq1$ (lower bound denoted by gray dashed line) rather than ${\frac{\sigma^{2}}{\sigma_{0}^{2}}\frac{T}{T_{0}}}/{\left( \frac{\kappa}{\kappa_{0}} \right)^{2}}>\frac{1}{2}$ (denoted by the black dashed line).

Supplementary Figure 3. The lower bound of ${\frac{\sigma^{2}}{\sigma_{0}^{2}}\frac{T}{T_{0}}}/{\left( \frac{\kappa}{\kappa_{0}} \right)^{2}}$ in 10D dynamics is also determined by the degree of non-gradient $\chi$ and the ratio between the two slowest timescales $T/{T_{2}}$, compared to Fig. 3 in the main text. (a, b) Numerical results of 10D systems show that the lower bounds derived in 2D (Eqs. (11) and (12) in the main text, dashed curve in (a) and (b), respectively) are valid for higher dimensions. The untight lower bound in (a) implies an overestimation of the degree of non-gradient, which is reasonable since the definition of $\chi$ depends on all dimensions of the dynamical systems, but the trajectory of which may only lie on a subspace in which the degree of non-gradient tends to be smaller.

Supplementary Figure 4. Numerical results of 2D and 10D dynamics with noisy feedback modules. (a, b) Numerical results of 2D (a) and 10D (b) dynamics, without the assumption that the feedback module does not introduce additional fluctuation, show that the trade-off (Eq. (8) in the main text) is still valid, but a clear shift toward larger noise direction (upward) appears, especially in high dimensional systems in which more additional fluctuation sources are introduced.

Supplementary Figure 5. Numerical results of 2D and 10D dynamics with increased fluctuation amplitude. (a, b) Numerical results of 2D (a) and 10D (b) dynamics, with relaxing the small noise condition through increasing the fluctuation amplitude by 10-fold, show that the trade-off (Eq. (8) in the main text) is still valid.
